# Aerobic Exercise Preserves Skeletal Muscle Function in Middle-Aged Mice Through the miR-150-5p/miR-199a-5p–Wnt/FZD4 Signaling Pathway

**DOI:** 10.3390/biology15131001

**Published:** 2026-06-25

**Authors:** Le Zhang, Jingzi He, Li Wang, Huan Zhang

**Affiliations:** 1 School of Physical Education, Yan’an University, Yan’an 716000, China; jingzihe1219@163.com; 2 School of Sports Medicine and Health, Chengdu Sport University, Chengdu 610041, China; wangli_cd001@163.com; 3 West China Medical Center, Sichuan University, Chengdu 610041, China

**Keywords:** aerobic exercise, middle-aged, sarcopenia, miRNA-seq, miR-199a-5p, miR-150-5p

## Abstract

MicroRNAs are important regulators of skeletal muscle development and regeneration; however, the molecular basis by which exercise-induced miRNAs preserve middle-aged muscle function remains to be elucidated. This study aimed to investigate how aerobic exercise delays skeletal muscle attenuation by reversing age-related miRNAs dysregulation in male mice. Twelve-month-old male C57BL/6J mice (MC) (*n* = 8/group) were randomly assigned to a sedentary control group (OC) or an aerobic exercise group (OE) (12 m/min, 40 min/session, three sessions/week, for 12 weeks). miRNA sequencing identified differentially expressed miRNAs (DEmiRNAs), followed by miRNA–mRNA network construction. The results demonstrated that aerobic exercise improved muscle strength and mass while attenuating early atrophy and fibrosis. Four atrophy-associated DEmiRNAs (miR-150-5p, miR-199a-5p, miR-3535, and miR-329-5p) were reversed after aerobic exercise intervention. GO and KEGG profiling demonstrated that target genes were predominantly involved in protein binding and the Wnt signaling pathway. miR-199a-5p and miR-150-5p, with the most predicted targets, were selected as candidate mechanistic contributors, and FZD4 was confirmed as a common downstream target. Further analysis confirmed that miR-199a-5p and miR-150-5p inhibition attenuated D-galactose-induced C2C12 myotube atrophy, reducing Atrogin-1 and increasing MyoD1, FZD4, and β-catenin expression. These findings suggest that the exercise-induced miR-150-5p/miR-199a-5p axis may alleviate muscle aging in middle age via the restoration of key proteins in Wnt signaling and contribute preliminary observational evidence relevant to the understanding of aerobic exercise intervention in sarcopenia.

## Simple Summary

Skeletal muscle mass and strength actually begin to decline during middle age, representing a critical window for sarcopenia intervention. This study investigated how aerobic exercise maintains skeletal muscle function at this stage through microRNAs. Twelve-month-old mice (human middle-age equivalent) were exercised (12 m/min, 40 min/session, three sessions/week, 12 weeks) or kept sedentary. Our results indicated that aerobic exercise attenuated muscle atrophy and fibrosis in middle-aged mice. By analyzing microRNA expression in skeletal muscle, we identified that miR-150-5p and miR-199a-5p may play important roles in this protective effect. Further experiments showed that inhibiting these microRNAs may help prevent myotube atrophy, possibly by restoring the expression of key proteins in the Wnt signaling pathway. These findings suggest that aerobic exercise may alleviate muscle aging in middle age by adjusting specific miRNAs and provide potential targets for early intervention against age-related sarcopenia.

## 1. Introduction

Sarcopenia, a degenerative age-related disorder manifesting through progressive myodegeneration and functional decline, represents a growing critical public health challenge [1]. Beyond impairing mobility and quality of life, it constitutes a common contributing factor for the etiology of diverse chronic conditions, spanning malignancy, diabetes mellitus, and osteoporosis [2,3,4], thereby imposing substantial socioeconomic and healthcare burdens [5]. While the reduction in skeletal muscle tissue initiates with an annual decline of roughly 1% after age 30, the rate of atrophy accelerates substantially at 65 years of age. By the eighth decade of life, cumulative muscle loss may approach nearly 53% [6,7,8]. However, previous studies have focused on the elderly stage, when skeletal muscle deterioration becomes evident, thereby overlooking the pre-symptomatic phase during which molecular and morphological alterations remain reversible. This late-stage intervention has yielded limited therapeutic efficacy, as pathological changes become largely irreversible once manifest [9,10]. Therefore, the period bridging middle and old age may represent a crucial window for preventing age-related skeletal muscle loss.

Aerobic exercise has been broadly acknowledged as one of the effective non-pharmacological strategies for preventing and managing sarcopenia, which exerts broad protective benefits for aged muscle by lowering oxidative stress levels as well as chronic inflammatory responses, boosting muscle protein synthesis while improving mitochondrial quality control and capillary density [11,12,13]. A previous study demonstrated that initiating moderate-intensity continuous training (MICT) at 9 months of age achieves superior improvements in the muscle stem cell regenerative capacity and an increase in the cross-sectional diameter and area of muscle fibers, coupled with a decrease in fibrosis when compared to initiation at 20 months in mice, suggesting that middle age may be a critical time window for exercise intervention to delay sarcopenia [14]. Therefore, the molecular mechanisms by which aerobic exercise in middle age delays sarcopenia warrant further investigation.

MicroRNAs are evolutionarily conserved, small non-coding RNAs that orchestrate post-transcriptional gene silencing through sequence-specific pairing with target mRNA molecules [15,16]. A growing body of evidence indicates that miRNAs serve as critical modulators in the molecular networks underlying skeletal muscle aging [17]. In aging models, miRNAs associated with muscle growth and regeneration are frequently downregulated, whereas those related to muscle wasting are often upregulated. These alterations may disrupt multiple signaling pathways, thereby contributing to the decline of skeletal muscle function [18]. Indeed, it has been well established that the alterations in miRNA expression triggered by exercise are critically involved in alleviating age-related muscle wasting [19,20,21]. Accordingly, understanding how middle-aged aerobic exercise modulates miRNA expression to preserve skeletal muscle function is of great importance.

Consequently, we hypothesized that aerobic exercise in middle age can counteract age-related muscle atrophy by regulating specific miRNAs and restoring key signaling pathways. Twelve-month-old mice (human middle-age equivalent) were subjected to 12 weeks of aerobic treadmill exercise in this study [22]. The safeguarding influence of middle-aged aerobic exercise against skeletal muscle deterioration was evaluated by assessing relative grip strength (body weight-normalized), together with relative gastrocnemius (body-weight-normalized), and the structural characteristics of myofibers. Furthermore, miRNA sequencing was performed to identify aerobic-exercise-induced miRNAs in skeletal muscle at middle age, subsequently subjected to GO and KEGG functional enrichment analyses to elucidate the molecular underpinnings. Key miRNA functions were further validated in a D-gal-induced C2C12 myotube atrophy model by cell immunofluorescence, qRT-PCR, and Western blotting. These findings may provide novel insights into the molecular pathogenesis of sarcopenia and offer a theoretical basis for the development of exercise mimetics against muscle atrophy associated with aging.

## 2. Materials and Methods

### 2.1. Animals and Treadmill Exercise Protocol

**Figure 1:**
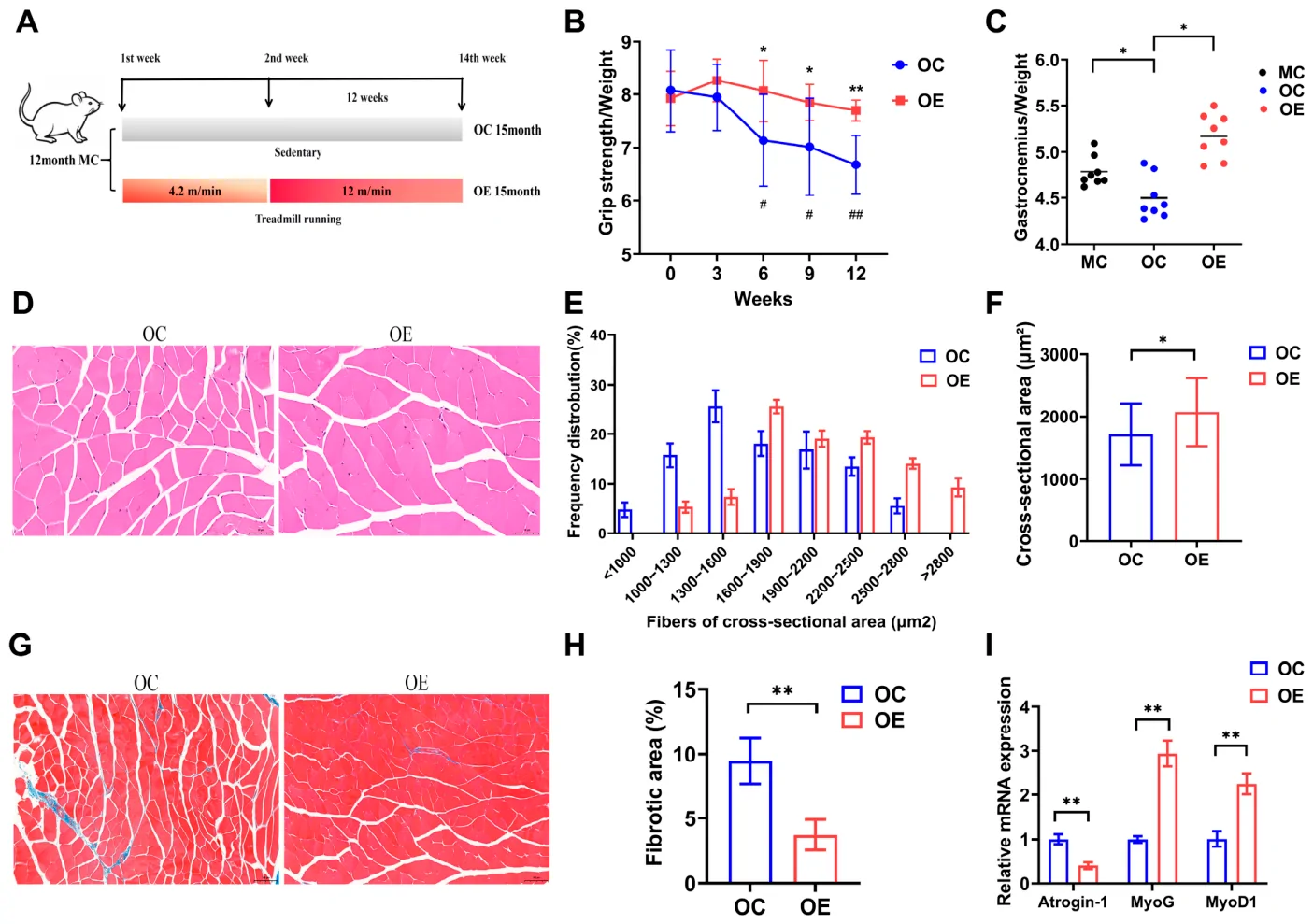
Aerobic exercise in middle age can delay the decline of mice’s skeletal muscle. (**A**) Schematic representation of animal ages, group assignments, and exercise intervention schedules. Twelve-month-old sedentary control group (early middle age, MC), 15-month-old sedentary control group (later middle age, OC), 12–15 months aerobic exercise group (OE). (**B**) The relative grip strength and (**C**) the relative gastrocnemius muscle mass in mice from the MC, OC, and OE groups (*n* = 8/group). (**D**) Representative HE-stained cross-sections of the gastrocnemius muscle in mice from the OC and OE groups. Scale bar = 50 μm. (**E**) Frequency distribution of myofiber CSA in the gastrocnemius muscle of OC and OE group mice (*n* = 6/group). (**F**) The average CSA of the gastrocnemius muscle myofibers in the OC and OE groups. (**G**) Representative histological sections stained with Masson’s trichrome of OC and OE mice. Scale bar = 100 μm. (**H**) Fibrotic area (as % of total area) in the gastrocnemius muscle of the OC and OE groups (*n* = 6/group). (**I**) qRT-PCR analysis of relative *Atrogin-1*, *MyoG* and *MyoD1* expression in the gastrocnemius muscle of the OC and OE groups. All results are shown as mean ± standard deviation (SD). * *p* < 0.05, ** *p* < 0.01 vs. OC group; # *p* < 0.05, ## *p* < 0.01 vs. MC group.

Male C57BL/6J mice (twelve months old, 32–35 g) were sourced from Beijing Vital River Laboratory (Beijing, China) and maintained in the SPF Animal Laboratory Center of Chengdu Sport University. Three mice per cage were maintained under standard conditions with adequate ventilation and free feeding. Following a seven-day acclimation period, the mice were divided into three groups using random assignment: a 12-month-old sedentary control group (*n* = 8, early middle age, MC), a 15-month-old sedentary control group (*n* = 8, later middle age, OC) and an aerobic treadmill running group at 12–15 months of age (*n* = 8, OE) (Figure 1A). Aerobic endurance training was performed using a non-weight-bearing treadmill running protocol outside the home cage, which included appropriate adjustments based on the condition of the mice, commencing at a starting treadmill velocity of 4.2 m per minute and zero grade (0° slope) for 1 week of adaptive training [21]. The formal running speed was set at 12 m/min for 40 min on a 0° slope, three times per week [23,24]. During the training, the three mice from the same home cage were trained simultaneously in three adjacent lanes to minimize isolation stress. We also monitored compliance by observing that all mice consistently ran without continuous encouragement, and any mouse that refused to run for >5 min on two consecutive sessions was removed (none occurred). The present investigation adhered rigorously to the National Regulations on Laboratory Animals (Ministry of Science and Technology, Beijing, China) throughout all phases of animal research. The study procedure was prospectively evaluated and formally endorsed by the Experimental Animal Ethics Committee of Chengdu University of Physical Education (Chengdu, China) [Adult Ethics (2022) No. 75] before any experimental procedures were undertaken. All animal-related activities, including acquisition, housing, care, and experimental manipulation, were performed by appropriately trained investigators in compliance with the approved protocol and institutional guidelines.

### 2.2. Assessment of Relative Grip Strength and Sarcopenia Index

During the 12-week period of aerobic exercise training, both grip strength and body weight were assessed at three-week intervals. The grip strength of the limbs was evaluated with a YLS-13A(Yiyan Technology, Jinan, China) apparatus for grip strength measurement. Each mouse was permitted to grip the grid with its limbs, during which a gentle backward pull was applied to the tail by the experimenter until the animal released the grid [25]. And three consecutive measurements were performed on each mouse, with the mean value being applied in the final analysis. Relative grip strength was calculated by grip strength normalized to body weight. Upon completion of the intervention, gastrocnemius muscles were harvested as the experimental objects and subsequently snap-frozen in liquid nitrogen and kept at −80 °C until further processing. The sarcopenia index was derived by gastrocnemius muscle mass (mg) normalized to body weight (g). The values were used to evaluate skeletal muscle attenuation in middle-aged mice (*n* = 8/group).

### 2.3. Histological Examination

The gastrocnemius muscles were preserved by overnight fixation at 4 °C using 4% paraformaldehyde (PFA). Twenty-four hours later, the tissues were processed by dehydration in 70% ethanol, subsequently processed for paraffin embedding and sectioning. For histological analysis, the resulting sections underwent deparaffinization, followed by staining with HE and Masson’s trichrome. The sections were observed under a Nikon ECLIPSE E100 light microscope (Nikon Corporation, Tokyo, Japan), and Image-Pro Plus 6.0 was employed for quantitative assessment. To evaluate fiber distribution, the CSA of approximately 100 myofibers was quantified in HE-stained sections, whereas Masson’s trichrome-stained sections served to determine the extent of muscle fibrosis (*n* = 6/group).

### 2.4. miRNA Sequencing

TRIzol reagent served to isolate total RNA from mice gastrocnemius muscle (MC, OC, and OE). The quality of extracted RNA was assessed jointly by a NanoDrop 2000 spectrophotometer (Thermo Scientific, Waltham, MA, USA) (for concentration and purity) and an Agilent 2100 Bioanalyzer (Agilent Technologies, Santa Clara, CA, USA) (for integrity). Lianchuan Biotechnology (Hangzhou, China) performed the preparation of small RNA libraries using the TruSeq Small RNA Prep Kit (Illumina, San Diego, CA, USA) and carried out sequencing on the HiSeq 2000/2500 platform (Illumina, San Diego, CA, USA), which produced 50 bp single-end reads. Data processing involved removing 3’ adaptors and non-target sequences, retaining sRNA with nucleotides from 18 to 26 bp, and filtering mismatches with mRNA, RFam and Repbase databases (excluding miRNA). The remaining reads were mapped to known miRNA precursors and the reference genome to identify miRNAs. DEmiRNAs were identified using DESeq2. The false discovery rate (FDR) was controlled by employing the Benjamini–Hochberg method. Selection criteria for DEmiRNAs were set as the absolute value of the fold difference in expression |log2FoldChange| > 1 with *p* < 0.05. TargetScan [26] and miRanda [27] were employed to identify the candidate potential target genes for DEmiRNAs. The overlapping results were designated as putative targets, which were subsequently analyzed by GO and KEGG enrichment methods.

### 2.5. Dual-Luciferase Reporter Assay

The synthetic mimics for miR-199a-5p and miR-150-5p, along with their corresponding negative control (NC), were sourced from GenePharma (Shanghai, China). To generate the reporter plasmids, the wild-type (WT) 3′UTR of Frizzled class receptor 4 (FZD4) harboring the predicted miRNA-binding sequence was inserted into the GP-miRGLO vector (GenePharma, Shanghai, China) via the SacI and XhoI restriction sites. Corresponding mutant (MUT) plasmids with altered seed-binding sequences were produced with a Fast Mutagenesis Kit (TransGen, Beijing, China). HEK293T cells were dispensed into 24-well plates and subjected to transfection upon reaching 70% confluence with 200 ng of WT or MUT reporter construct and 50 nM miRNA mimic (miR-150-5p/miR-199a-5p) or mimic NC using Lipofectamine 3000 (Invitrogen, Carlsbad, CA, USA). Transfected cells were harvested at the 48 h time point, and luciferase activity was subsequently evaluated using the Dual Luciferase Assay Kit (GenePharma, Shanghai, China) in line with the recommended procedure. For data normalization, the relative reporter activity was expressed as firefly/Renilla luciferase luminescence ratios.

### 2.6. Cell Culture and Experimental Interventions

C2C12 cells were cultured in complete growth medium comprising high-glucose DMEM, 10% fetal bovine serum, and 1% penicillin–streptomycin (Servicebio, Wuhan, China), with incubation at 37 °C in a 5% CO_2_ humidified incubator. Cells received regular medium changes and were subcultured when they reached 70–80% confluence. For differentiation, C2C12 cells were placed into 6-well plates with a density of 2 × 10^5^ cells per well. Once the cells reached nearly complete confluence (90–100%), the culture medium was switched to differentiation medium (supplemented with 2% horse serum), and the cells were incubated to undergo a 4-day differentiation process to generate myotubes. Lipofectamine 3000 (Invitrogen, Carlsbad, CA, USA) was used to transfect the differentiated C2C12 cells with inhibitors targeting miR-199a-5p, miR-150-5p, or a negative control (inhibitor NC). The culture medium was refreshed at 6 h after transfection, and incubation continued for an additional 18 h. Thereafter, 40g/L D-galactose was administered at the selected working concentration based on our pilot dose–response experiments and previous reports to induce myotube atrophy for 48 h [28].

### 2.7. Immunofluorescence Staining

To evaluate myotube morphology, the C2C12 cells were subjected to three PBS rinses and subsequently fixed in 4% paraformaldehyde for 15 min at room temperature. Cellular membranes were rendered permeable by treatment with 0.3% Triton X-100 in PBS for 10 min. After three additional PBS washes, cells were blocked with 5% BSA in PBS for 1 h prior to primary antibody incubation. The prepared samples were placed under incubation for 16 h at 4 °C with anti-myosin 4 monoclonal antibody conjugated to Alexa Fluor 488 (MF20; 1:100; Cat# 53-6503-82, Invitrogen, Carlsbad, CA, USA). On the following day, the cells underwent three washes with PBS supplemented with 0.1% Tween-20. Nuclear staining was achieved via 5 min incubation with DAPI (Solarbio, Beijing, China). Fluorescence images were acquired with an Olympus IX73 fluorescence microscope (Olympus, Tokyo, Japan). The quantification of myotube fusion index was performed using ImageJ software (version 1.54p).

### 2.8. Quantitative Reverse Transcription PCR (qRT-PCR)

TRIzol reagent (Solarbio, Beijing, China) was employed to extract total RNA from mice’s gastrocnemius muscle tissues and cultured C2C12 cells. For mRNA analysis, cDNA synthesis was conducted employing ABScript RT Master Mix, and qRT-PCR was subsequently carried out with SYBR Green Fast qPCR Mix (ABclonal, Wuhan, China) on a real-time PCR platform. For miRNA analysis, complementary DNA and qRT-PCR were performed using the Hairpin-it miRNAs Kit (GenePharma, Shanghai, China). GAPDH together with β-actin served as endogenous controls to normalize the expression levels of target mRNAs, while U6 served as the reference gene for miRNA quantification. Primer sequences are listed in Table S1. Relative mRNA (or miRNA) levels were determined by the 2^−ΔΔct^ quantification method. Experimental procedures were replicated independently three times using distinct biological samples, and each assay was conducted in triplicate.

### 2.9. Western Blot

The cellular proteins were extracted using RIPA lysis buffer (Servicebio, Wuhan, China) with protease and phosphatase inhibitors and quantified by BCA assay (Servicebio, Wuhan, China). Samples were heat-denatured at 95 °C for 5 min in the presence of SDS loading buffer, separated by molecular weight on 8–12% SDS-polyacrylamide gels, and transferred onto PVDF membranes using a wet tank transfer system. After transfer, the membranes were subjected to a 1 h blocking step (room temperature), followed by 4 °C overnight incubation with the primary antibody: rabbit polyclonal antibody anti-FBXO32 (1:1000; ABclonal), anti-MYOD1 (1:1000; Invitrogen), anti-β-catenin (1:1000; Proteintech, Shanghai, China), anti-FZD4 (1:500; ABclonal), anti-AXIN2(1:1000; ABclonal), anti-Cyclin D1 (1:1000; Selleck, Shanghai, China) and anti-β-actin (1:1000; Servicebio) as the endogenous loading control. Membranes were rinsed thrice with TBST prior to secondary antibody application, followed by incubation with species-matched HRP-conjugated secondary antibodies (1:10,000; Proteintech) for 60 min at ambient temperature. Immunoreactive signals were developed with an ECL reagent (Servicebio) and subsequently recorded using a ChemiDoc MP imaging platform (Bio-Rad, Hercules, CA, USA). Densitometric quantification was calculated using ImageJ software (version 1.54p).

### 2.10. Statistical Analysis

All experimental data analysis and graphical presentation were carried out in GraphPad Prism v10.0. Statistical significance between two groups was assessed by unpaired two-tailed Student’s *t*-test. For three groups (YC, OC, and OE), we used one-way ANOVA followed by Tukey’s post hoc test. ns *p* > 0.05 indicated no significant difference, whereas * *p* < 0.05 and ** *p* < 0.01 represented significant and highly significant differences, respectively.

## 3. Results

### 3.1. Effects of Aerobic Exercise Intervention on Skeletal Muscle Attenuation in Middle-Aged Mice

In contrast to the MC group, the OC group exhibited a marked reduction in both relative grip strength (*p* < 0.01) and relative gastrocnemius muscle mass (*p* < 0.05). However, the OE group showed a significant increase in both parameters when set against the OC group (*p* < 0.01 for relative grip strength, *p* < 0.05 for relative gastrocnemius muscle mass) (Figure 1B,C). To better observe the influence of middle-aged aerobic exercise intervention on muscle morphology in middle-aged mice, we performed HE and Masson’s trichrome staining on gastrocnemius muscle. In contrast to the OC group, the OE group had more uniformly distributed gastrocnemius muscle fibers with a larger average CSA based on HE-stained observations (Figure 1D–F). Furthermore, gastrocnemius muscle fibrosis was significantly mitigated in the OE group (Figure 1G,H), accompanied by decreased *Atrogin-1* and increased *MyoG* and *MyoD1* expression (Figure 1I).

### 3.2. Identification of DEmiRNAs in Middle-Aged Skeletal Muscle Under Aerobic Exercise Intervention

**Figure 2:**
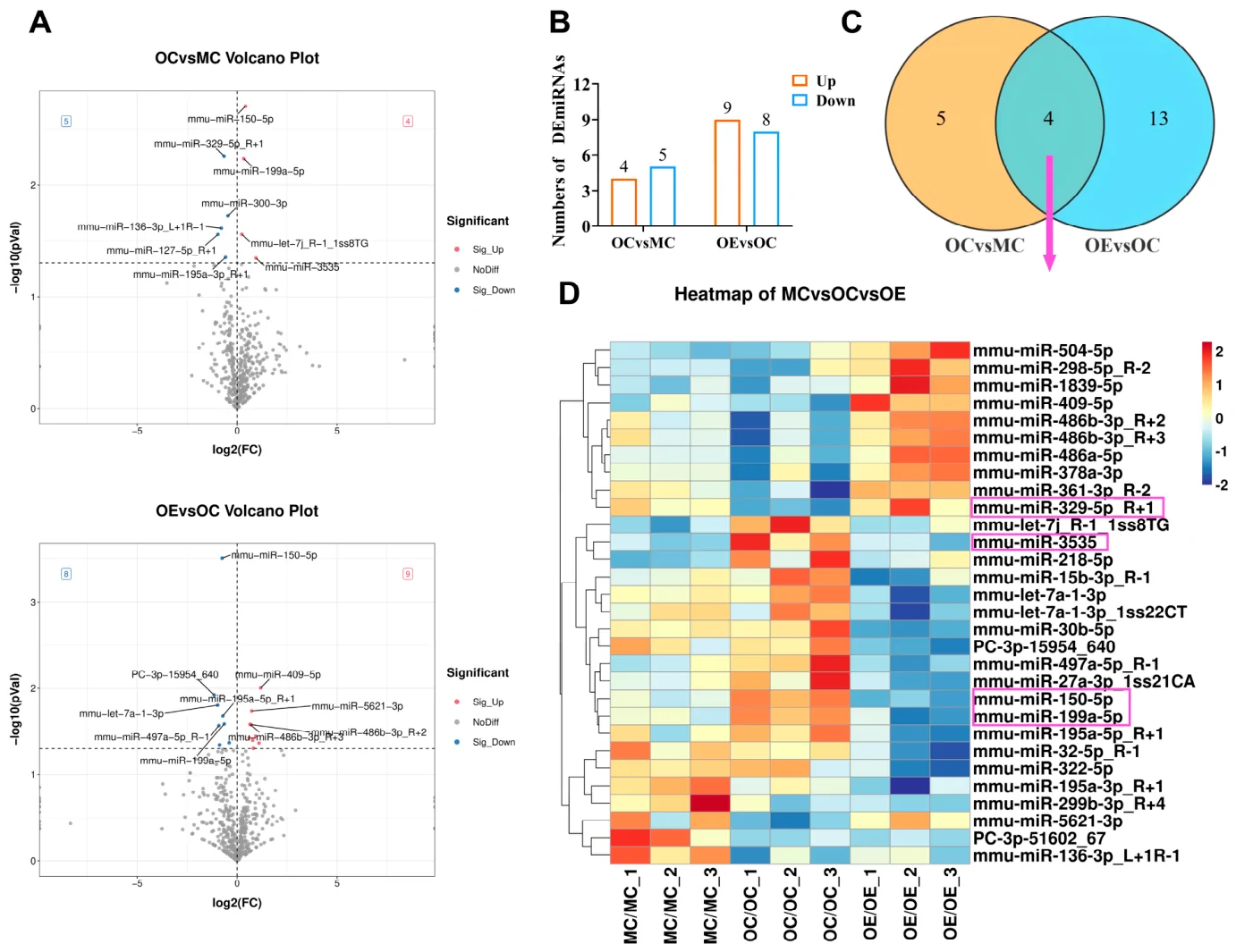
Hierarchical Clustering of DEmiRNAs in gastrocnemius muscle of middle-aged mice. (**A**) The volcano plots illustrating DEmiRNAs. Upper panel: OC vs. MC; lower panel: OE vs. OC. (upregulated miRNAs: red; downregulated miRNAs: blue). (**B**) Vertical histogram quantifying the number of significantly upregulated and downregulated DEmiRNAs in each group contrast. (**C**) Venn diagram depicting shared and unique DEmiRNA subsets across the age-related (OC vs. MC) and exercise-responsive (OE vs. OC) comparisons. (**D**) The heatmap of DEmiRNAs in skeletal muscle of middle-aged mice (red: high, blue: low).

There were nine and 17 miRNAs with significantly differential expression (|log2FC| ≥ 1.5, *p* < 0.05) in the OC group, following separate comparisons with the MC and OE groups, and a cluster analysis of the expression profiles was subsequently conducted (Figure 2A). The DEmiRNA expression patterns in each group were visualized using vertical histograms and heatmaps. The OC vs. MC group showed four upregulated and five downregulated miRNAs, while the OE vs. OC group showed nine upregulated and eight downregulated miRNAs. (Figure 2B). Integrated analysis of the two comparison groups showed that four miRNAs reversed after aerobic exercise intervention, including miR-150-5p, miR-199a-5p, miR-3535 and miR-329-5p_R+1 (Figure 2C,D).

### 3.3. The GO and KEGG Functional Enrichment Analysis

**Figure 3:**
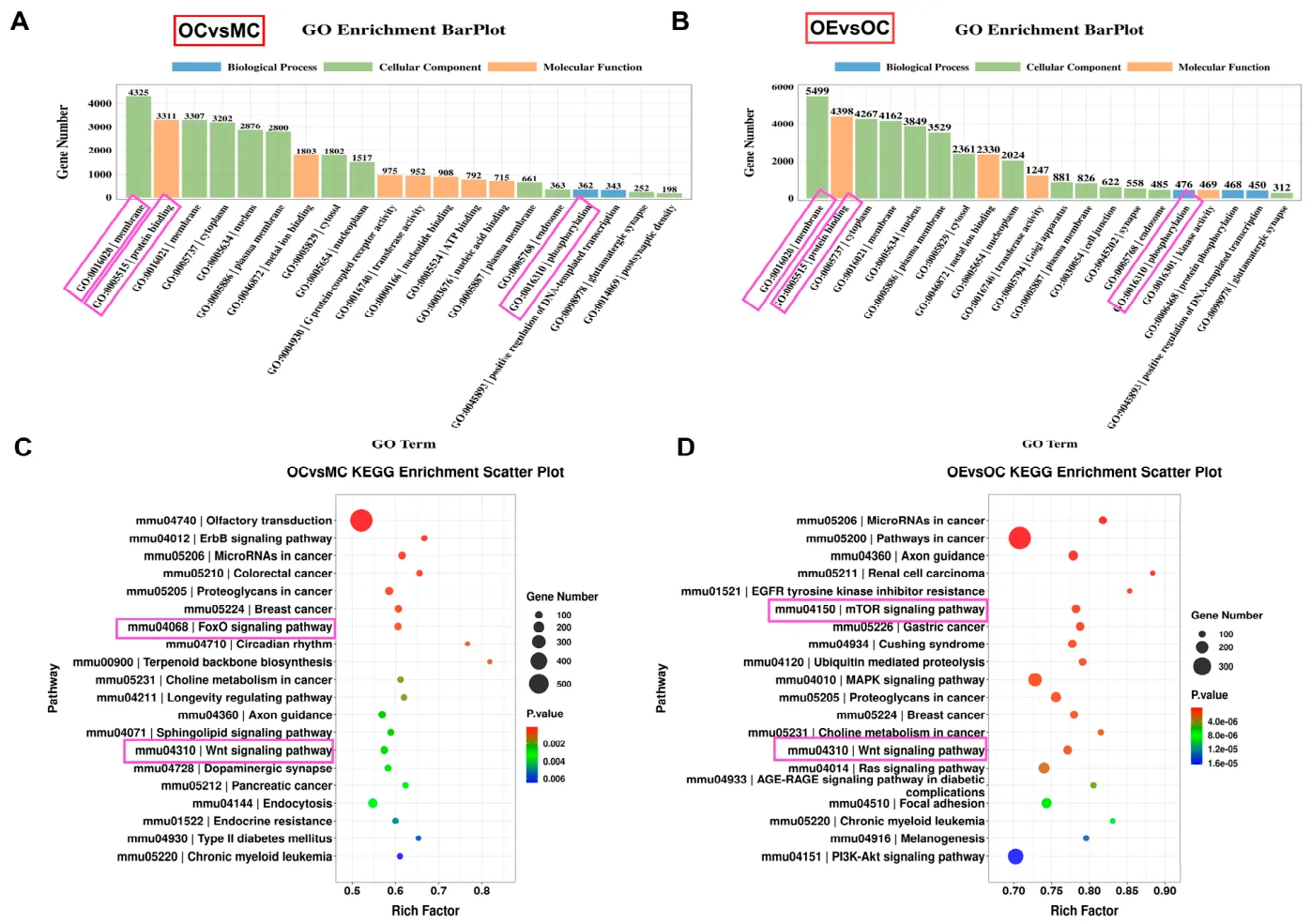
The GO and KEGG functional annotation and enrichment analysis. (**A**,**B**) GO enrichment analysis of DEmiRNA target Genes: age-related (OC vs. MC) and exercise-responsive (OE vs. OC) comparisons. (**C**,**D**) KEGG pathway enrichment of DEmiRNA target genes: age-related (OC vs. MC) and exercise-responsive (OE vs. OC) comparisons. Circle size scales with gene abundance, and color depth corresponds to the magnitude of statistical significance (*p*-value). The boxed regions indicate the enrichment terms and pathways of primary interest in this study.

The predicted target genes were subjected to systematic functional annotation via GO term and KEGG pathway analysis aimed at identifying functional enrichments of these DEmiRNAs. The target genes in the OC vs. MC group were mainly enriched in phosphorylation (BP), membrane (CC) and protein binding (MF) (Figure 3A), and the same result also occurred in the OE vs. OC group (Figure 3B). Comprehensive analysis indicated that the KEGG pathway enrichments for the target genes of DEmiRNAs in the OC vs. MC group were Foxo and Wnt signaling pathways (Figure 3C), while in the OE vs. OC group, they were mTOR and Wnt signaling pathways (Figure 3D).

### 3.4. Validation of miRNA Sequencing Data

**Figure 4:**
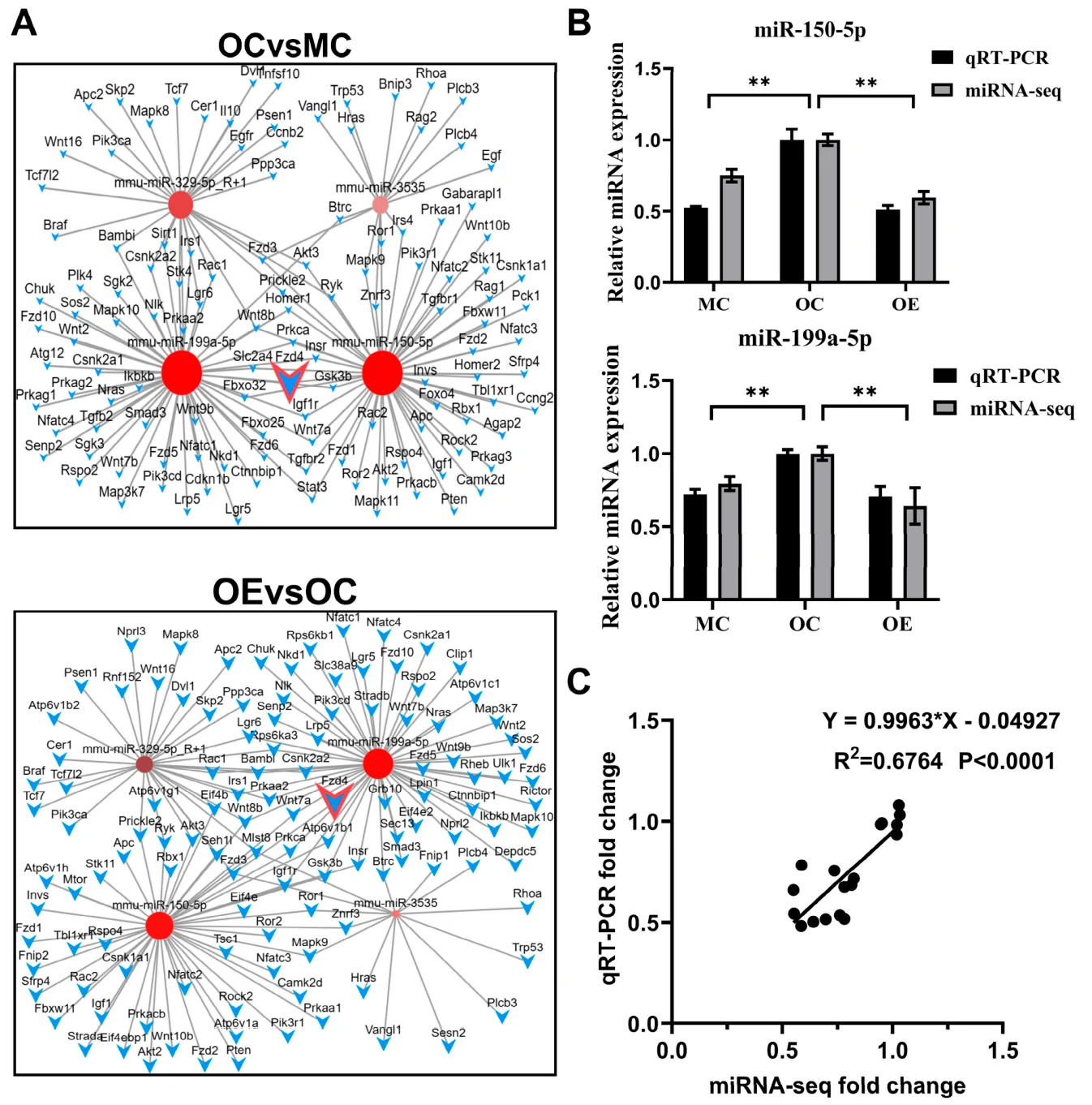
Validation of miRNA-seq data in gastrocnemius muscle of middle-aged mice. (**A**) Illustration of the miRNA–mRNA interaction network linked to the Wnt and FoxO pathways (OC vs. MC) and to the Wnt and mTOR pathways (OE vs. OC)( Red circles represent miRNAs, with larger circles indicating a greater number of predicted target genes. Blue arrows denote target genes). (**B**) qRT-PCR was carried out on DEmiRNAs to validate their differential expression. (**C**) Pearson correlation analysis was performed between the qRT-PCR and miRNA-seq results. R^2^ represents the correlation coefficient; the Pearson correlation analysis yielded a *p* < 0.0001. All results are shown as mean ± SD. ** p < 0.01 vs. OC group.

The bioinformatically predicted miRNA–mRNA interaction illustration (four reversed miRNAs corresponding to 273 target genes in Foxo and Wnt signaling pathways (OC vs. MC) or 293 target genes in mTOR and Wnt signaling pathways (OE vs. OC)) is depicted (Figure 4A). The differential expression of DEmiRNAs was subjected to qRT-PCR validation. In the OC vs. MC group, miR-150-5p, as well as miR-199a-5p (characterized by more target genes and higher RPM (Reads Per Million) values), demonstrated a significant upregulation (Supplementary File S2). Aerobic exercise intervention rescued the changes in these miRNAs caused by aging (Figure 4B). Additionally, the precision of miRNA expression levels was supported by the concordance between qRT-PCR and miRNA-seq analyses (*p* < 0.0001; Figure 4C).

### 3.5. miR-199a-5p/miR-150-5p Inhibition Ameliorate D-Gal-Induced C2C12 Myotube Atrophy

**Figure 5:**
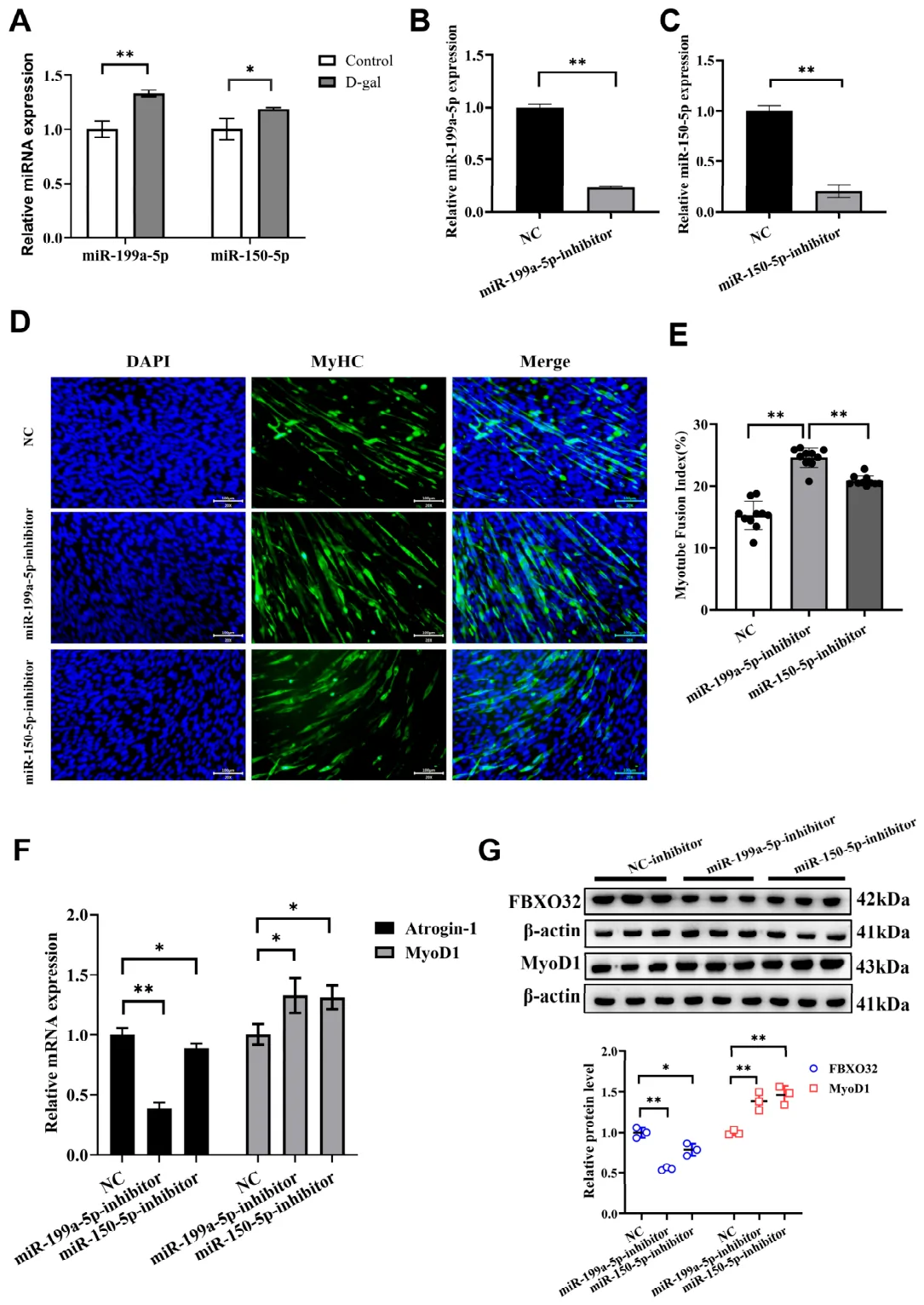
The transfection of miR-199a-5p/miR-150-5p inhibitors ameliorates D-gal-induced C2C12 myotube atrophy. (**A**) qRT-PCR analysis of relative miR-199a-5p or miR-150-5p expression in D-gal-treated C2C12 myotubes, normalized to the untreated control group. (**B**,**C**) qRT-PCR quantification of miR-199a-5p and miR-150-5p expression, respectively, following transfection with specific inhibitors in D-gal-treated C2C12 myotubes. (**D**) Representative immunofluorescence micrographs of MyHC (myosin heavy chain) staining in D-gal-treated C2C12 myotubes following the transfection of NC, miR-199a-5p and miR-150-5p inhibitors. Scale bars = 100 µm. (**E**) The quantification of myotube fusion in D-gal-treated C2C12 myotubes following the transfection of miR-199a-5p or miR-150-5p inhibitors relative to the NC inhibitor group. Each data point represents the mean of 10 random microscopic fields per well. (**F**) qRT-PCR analysis of relative *Atrogin-1* and *MyoD1* mRNA expression in D-gal-treated C2C12 myotubes following the transfection of miR-199a-5p or miR-150-5p inhibitors relative to the NC inhibitor group. (**G**) Western blot analysis of relative FBXO32 and MyoD1 protein expression in D-gal-treated C2C12 myotubes following the transfection of miR-199a-5p or miR-150-5p inhibitors relative to the NC inhibitor group. * p < 0.05 and ** p < 0.01 indicate a statistically significant change relative to the NC inhibitor group.

qRT-PCR analysis revealed that D-gal treatment significantly upregulated miR-199a-5p and miR-150-5p expression in C2C12 myotubes compared to untreated controls (Figure 5A). The knockdown efficiency of miR-199a-5p and miR-150-5p reached approximately 80% in C2C12 myotubes transfected with specific inhibitors (*p* < 0.01) (Figure 5B,C). In particular, the fusion index of myotubes was significantly increased to 24.55 ± 1.57% and 20.86 ± 0.80% with the transfection of miR-199a-5p and miR-150-5p inhibitors, respectively, compared to NC inhibitors (15.24 ± 2.26%) in D-gal-induced C2C12 myotube atrophy (Figure 5D,E, *p* < 0.01). In addition, we observed that *MyoD1* (a myogenic factor) expression was significantly increased, whereas *Atrogin-1* (FBXO32, a muscle atrophy-associated factor) was significantly downregulated with the transfection of miR-199a-5p or miR-150-5p inhibitors in D-gal-treated C2C12 myotubes (Figure 5F,G, Supplementary File S1).

### 3.6. miR-199a-5p/miR-150-5p Modulate Myotube Atrophy by Restoring the Key Proteins in Wnt Pathway

**Figure 6:**
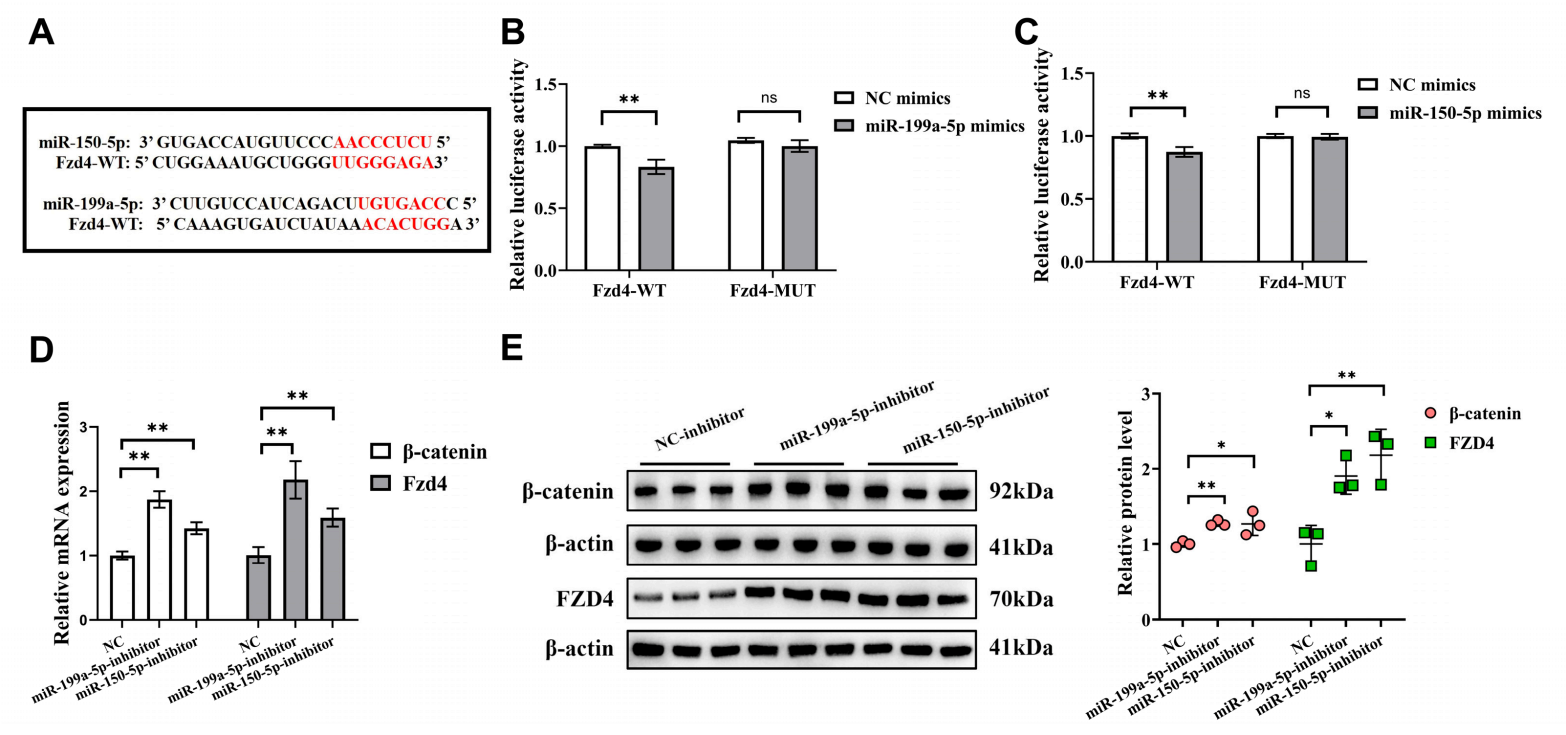
miR-199a-5p and miR-150-5p regulate muscle atrophy through the restoration of key proteins in the Wnt pathway. (**A**) Complementary sequences to the seed regions of miR-199a-5p/miR-150-5p within Fzd4 3’-UTR. (**B**,**C**) The relative luciferase activity in cells co-transfected with miR-199a-5p/miR-150-5p mimics or NC mimics and the WT or MUT Fzd4 3’-UTR reporter vectors. Each experiment was performed in triplicate. Renilla luciferase activity served as the internal normalization control. (**D**) qRT-PCR analysis of relative *Fzd4* and *β-catenin* mRNA expression following the transfection of miR-199a-5p or miR-150-5p inhibitors in comparison with the NC inhibitor group. (**E**) Western blot quantification of Fzd4 and β-catenin protein expression following the transfection of miR-199a-5p or miR-150-5p inhibitors in comparison with the NC inhibitor group. All data are relative to housekeeping gene expression and represent mean ± SD (*n* ≥ 3). * p < 0.05 and ** p < 0.01 indicate a statistically significant change relative to the NC inhibitors group. ns (not significant, *p* > 0.05).

The putative binding positions of miR-199a-5p/miR-150-5p on the 3’-UTR of FZD4 are presented in Figure 6A. In the dual-luciferase reporter system, co-transfection with miR-199a-5p mimics and FZD4 wild-type (WT) plasmid led to a pronounced reduction in luciferase activity relative to co-transfection with NC mimics and FZD4-WT plasmid (*p* < 0.01). On the contrary, there was no statistically significant difference in luciferase activity following co-transfection with either the NC mimics or the miR-199a-5p mimics together with the mutant (MUT) FZD4 plasmid (*p* > 0.05) (Figure 6B). A comparable outcome was seen with the miR-150-5p and FZD4 interaction (Figure 6C). Relative to the NC inhibitors group, transfection with miR-199a-5p and miR-150-5p inhibitors in D-gal-treated C2C12 myotubes significantly increased FZD4 and β-catenin expression (Figure 6D,E, Supplementary File S1). Concurrently, expression levels of the canonical Wnt-responsive genes Axin2 and Cyclin D1 were also upregulated (Figure S1).

## 4. Discussion

Sarcopenia represents an age-related myodegenerative disorder hallmarked by progressive deterioration of muscle mass and function, profoundly undermining physical independence and quality of life in senescent individuals. Exercise can prevent and alleviate aging-induced muscle dysfunction. However, research on aerobic intervention in aging-related skeletal muscle diseases by exercise focuses on the onset of aging. The research on aerobic exercise in 18-month-old mice revealed that with the occurrence of osteoporosis, skeletal muscle function deteriorates alongside aging, which seriously restricts the exercise intensity that the mice can withstand [23]. Therefore, we hypothesized that starting exercise in middle age might improve the exercise adaptability of skeletal muscle to better cope with the age-related decline in exercise capacity. The 12-month middle-aged mice, equivalent to 38–47-year-old humans, were selected as the study object. Notably, the relative grip strength (Figure 1B) and the relative gastrocnemius muscle mass (Figure 1C) in the OC group exhibited significantly lower values relative to the MC group, suggesting the onset of skeletal muscle aging [29]. After a 12-week aerobic exercise intervention, the OE group exhibited significant increases in both parameters. Meanwhile, a more uniform arrangement of muscle fibers was observed, accompanied by an increase in average CSA (Figure 1D–F). In contrast, the fibrotic area in OE mice was markedly smaller than that in OC mice (*p* < 0.01). Additionally, the downregulation of skeletal muscle atrophy markers *Atrogin-1* (Fbxo32), coupled with the upregulation of myogenic regulators *MyoD1* and *MyoG*, suggests a phenotypic shift from muscle catabolism to anabolism (Figure 1I). These findings provide preliminary evidence that 12 weeks of aerobic exercise may help preserve skeletal muscle function in middle-aged mice. Although starting exercise in old age is still effective, beginning long-term aerobic exercise in middle age can bring about more lasting benefits, suggesting the importance of preventive intervention [14]. Middle age is a period of gradual decline in physiological function, and studying the intervention effects and mechanisms of exercise at this stage can provide a scientific basis and strategies for health security in old age.

Aging is associated with a specific dysregulation of the miRNA network, whereas exercise training rebalances these opposing signals, thereby suppressing catabolic pathways and enhancing protein synthesis signaling [20,21,30]. In this study, nine DEmiRNAs were identified to be related to muscle loss during midlife in the OC vs. MC group (Figure 2A). After aerobic exercise intervention, 17 DEmiRNAs were found in the OE vs. OC group (Figure 2B). The intersection of the comparison groups yielded four miRNAs (miR-150-5p, miR-199a-5p, miR-3535 and miR-329-5p_R+1) that might be reversed after middle-aged aerobic exercise intervention (Figure 2C,D). GO enrichment analysis provided important functional annotation for predicted target genes. Enriched terms such as phosphorylation (BP), membrane (CC) and protein binding (MF) suggest that exercise-reversed miRNAs may participate in the regulation of complex molecular interaction networks rather than acting through a single downstream effector (Figure 3A,B). This was not consistent with previous studies; after 12 weeks of exercise intervention in aged rats, the predicted targets of DEmiRNAs showed significant enrichment in apoptosis and autophagy pathways [21], which fully demonstrates the differences between aerobic exercise in middle age and that in old age, with an increased emphasis on the recovery of skeletal muscle function. Additionally, KEGG analysis further suggested that the Wnt signaling pathway may be modulated by midlife aerobic exercise (Figure 3C,D). Previous studies demonstrated that the Wnt pathway may be involved in the complex and crucial modulation of sarcopenia. In aged skeletal muscle, overactivation of Wnt signaling may induce myofibroblast conversion from satellite cells, suggesting a mechanism that could compromise regenerative capacity [31]. In contrast, the upregulation of Wnt signaling triggered by exercise accelerates Myf5 and MyoD gene transcription, thereby converting satellite cells into an activated state and increasing muscle regenerative capacity [32]. We postulate that aerobic exercise in middle age may restore the key proteins in the Wnt/β-catenin pathway through miRNA-mediated regulation, preventing pathological overactivation while preserving physiological signaling. Therefore, we focused on miR-199a-5p and miR-150-5p, which have more candidate target genes enriched in the Wnt signaling pathway (Figure 4A). In addition, sequencing results showed that the RPM (Reads Per Million) values of miR-3535 and miR-329-5p in the gastrocnemius muscle were significantly lower than those of miR-150-5p and miR-199a-5p (Supplementary File S2). And qRT-PCR results showed that miR-3535 and miR-329-5p have very low baseline expression in gastrocnemius muscle samples (Ct > 35), making functional manipulation challenging. It is also worth noting that no studies have been reported regarding the roles of miR-3535 and miR-329-5p in muscle, while some studies have indicated that miR-199a-5p, as a fibromiR, is upregulated in Duchenne Muscular Dystrophy [33,34]. Another study has found that adipose tissue in obese individuals can specifically induce primary human myotube atrophy in the elderly by secreting extracellular vesicles carrying miR-150-5p. And the myotube atrophy induced by exosomes can be significantly alleviated, and the thickness of myotubes can be restored by the downregulation of miR-150-5p [35]. In our study, miR-199a-5p and miR-150-5p showed elevated expression in the OC group when compared with the MC group, while they decreased significantly after aerobic exercise intervention (Figure 4B), which provides additional evidence that miR-199a-5p and miR-150-5p could be potential candidate exercise-induced miRNAs.

**Figure 7:**
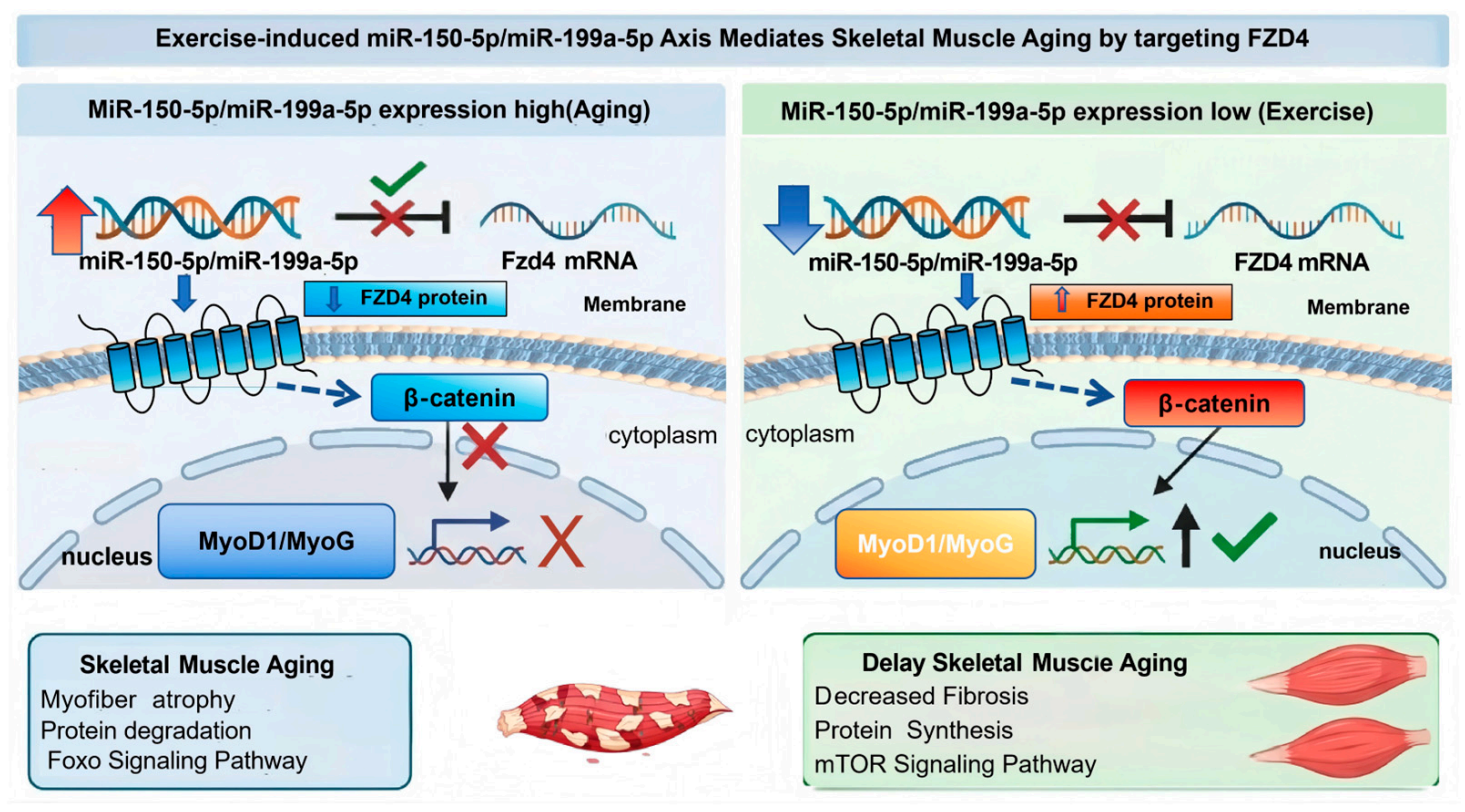
A speculative diagram of the potential mechanism by which aerobic-exercise-responsive miRNAs may help maintain skeletal muscle function in middle-aged mice. A check mark (✓) denotes restoration or functional recovery, a cross mark (✗) denotes inhibition or suppressive effects.

To gain mechanistic insight into how miR-199a-5p and miR-150-5p sustain skeletal muscle homeostasis in middle-aged mice, we modeled C2C12 cell aging by 40 g/L D-gal based on our pilot dose–response experiments and previous reports in vitro [36]. Although this model cannot fully recapitulate muscle atrophy occurring during natural aging, which involves multiple factors (such as neuromuscular junction degeneration, chronic inflammation, hormonal alterations, and mitochondrial dysfunction), the D-gal-induced C2C12 myotube atrophy model nevertheless effectively simulates skeletal muscle atrophy resulting from oxidative stress accumulation during natural aging [37]. Its relatively low culture cost and exemption from animal ethics approval cycles make it an effective and convenient in vitro tool for investigating the molecular mechanisms of aging-related muscle atrophy that is particularly suitable for screening candidate molecules and preliminarily validating signaling pathways [38,39]. Our findings revealed that D-gal treatment elicited robust upregulation of miR-150-5p and miR-199a-5p, consistent with our observations in middle-aged mice (Figure 5A). Functionally, miR-150-5p and miR-199a-5p inhibition effectively attenuated D-gal-induced C2C12 myotube atrophy (Figure 5D,E). From the overlapping predicted targets of miR-150-5p and miR-199a-5p, we prioritized FZD4 for further investigation (Figure 6A). More importantly, Wnt proteins typically initiate downstream signaling through binding to Frizzled receptors on the cell membrane, which is consistent with the results obtained from GO terms and KEGG pathway analysis (Figure 3). There is evidence to suggest that FZD4 could play a positive role during muscle development. For instance, FZD4 overexpression appeared to mitigate the miR-136-5p-mediated suppression of C2C12 myoblast proliferation and differentiation [40]. Additionally, in a rat muscle contusion model, the expression of FZD4 was markedly elevated at the early stage after injury and then gradually declined, a pattern consistent with that of MyoD expression [41]. In this study, the restoration of FZD4 and β-catenin expression was accompanied by increased transcription of Axin2 and Cyclin D1 (Figure S1). Both Axin2 and Cyclin D1 are recognized as key downstream transcriptional targets of the canonical Wnt signaling pathway [42,43]. Upon Wnt signaling activation, their expression is upregulated. However, the subsequent increase in Axin2 expression potentially contributes to limiting excessive signal activation through negative feedback, thereby indirectly influencing Cyclin D1 expression level [44,45]. These observations raise the possibility that limiting excessive Wnt signaling activation may become a potential strategy for preventing and treating muscle atrophy. Collectively, our results raise the possibility that the miR-150-5p/miR-199a-5p–FZD4 axis may serve as a candidate regulatory mechanism of exercise intervention against sarcopenia, as identified through bioinformatic prediction and in vitro validation (Figure 7), though it should not be interpreted as the confirmed primary mechanism underlying the protective effects of exercise in vivo. Accordingly, the conclusions drawn from this model warrant further validation in in vivo experiments or clinical samples. Additionally, as this investigation only utilized male mice, the results might not be fully applicable to females. Future investigations should therefore include both sexes to explore potential sex-specific responses to aerobic exercise in the context of sarcopenia.

## 5. Conclusions

This study offers preliminary evidence that 12 weeks of aerobic exercise exerts beneficial effects on the functional capacity of skeletal muscle in middle-aged male mice, partly through the reversal of age-related miRNA dysregulation. As the candidate mechanistic contributors, the inhibition of miR-150-5p and miR-199a-5p may alleviate myotube-atrophy-associated phenotypes by converging on FZD4, a common downstream effector of the Wnt/β–catenin signaling axis. Collectively, these experimental outcomes provide mechanistic insights into how aerobic exercise attenuates skeletal muscle decline during middle age and provide a rationale for developing early intervention strategies against sarcopenia.

## Data Availability

The raw sequencing data generated in this study are publicly available in the NCBI SRA database under accession numbers [PRJNA1298960] (https://www.ncbi.nlm.nih.gov/sra/PRJNA1298960) (accessed on 21 June 2026) and [PRJNA1456757] (https://www.ncbi.nlm.nih.gov/sra/PRJNA1456757) (accessed on 21 June 2026) (Dataset: “miRNA raw data of mice gastrocnemius muscles under middle-aged aerobic exercise” and “miRNA raw data of middle-aged mice gastrocnemius muscles under aerobic exercise”).

## Supplementary Materials

The following supporting information can be downloaded at: https://www.mdpi.com/article/10.3390/biology15131001/s1, Supplementary File S1: WB Source Data; Supplementary File S2: The differentially expressed miRNAs in MC, OC and OE groups. Figure S1: miR-199a-5p and miR-150-5p regulates muscle atrophy through the restoration of key protein in Wnt pathway. (**A**) The relative mRNA expression levels of Axin2 and Cyclin D1 following the transfection of miR-199a-5p or miR-150-5p inhibitors in comparison with NC inhibitors group. (**B**) The relative protein expression levels of Axin2 and Cyclin D1 following the transfection of miR-199a-5p or miR-150-5p inhibitors in comparison with NC inhibitors group. All results are shown as mean±SD. * *p* < 0.05 and ** *p* < 0.01 indicate a statistically significant change relative to the NC inhibitors group; Table S1: Primer sequence for qRT-PCR.

## Abbreviations

The specialized terms appearing in abbreviated form herein are expanded below:

SPF	Specific-Pathogen-Free
MC	Sedentary control group (12 months old)
OC	Older Sedentary control group (15 months old)
OE	Aerobic exercise group (12–15 months old)
CSA	Cross-Sectional Area
HE	Hematoxylin and Eosin
D-gal	D-galactose
3′UTR	3′-untranslated region
RPM	Reads Per Million
WT	wild-type
MUT	mutant
