# Correction: Zhang et al. Aerobic Exercise Preserves Skeletal Muscle Function in Middle-Aged Mice Through the miR-150-5p/miR-199a-5p–Wnt/FZD4 Signaling Pathway. *Biology* 2026, *15*, 1001

**DOI:** 10.3390/biology15171475

**Published:** 2026-09-01

**Authors:** Le Zhang, Jingzi He, Li Wang, Huan Zhang

**Affiliations:** 1 School of Physical Education, Yan’an University, Yan’an 716000, China; jingzihe1219@163.com; 2 School of Sports Medicine and Health, Chengdu Sport University, Chengdu 610041, China; wangli_cd001@163.com; 3 West China Medical Center, Sichuan University, Chengdu 610041, China

## Text Correction

There was a factual error in the original paper [1] regarding the citation of reference ‘[35]’ in the Discussion section.

As such, a correction has been made to Section 4, paragraph 2. The corrected text now reads: “Another study has found that adipose tissue in obese individuals can specifically induce primary human myotube atrophy in the elderly by secreting extracellular vesicles carrying miR-150-5p. And the myotube atrophy induced by exosomes can be significantly alleviated, and the thickness of myotubes can be restored by the downregulation of miR-150-5p [35]”.

The authors state that the scientific conclusions are unaffected. This correction was approved by the Academic Editor. The original publication has also been updated.

## References

[1] Zhang L., He J., Wang L., Zhang H. (2026). Aerobic Exercise Preserves Skeletal Muscle Function in Middle-Aged Mice Through the miR-150-5p/miR-199a-5p–Wnt/FZD4 Signaling Pathway. Biology.

