# Supplementary material for: Aerobic Exercise Preserves Skeletal Muscle Function in Middle-Aged Mice Through the miR-150-5p/miR-199a-5p–Wnt/FZD4 Signaling Pathway
Source: Biology (Basel). 2026 Jun 25;15(13):1001. doi: 10.3390/biology15131001 (PMC13359755; doi:10.3390/biology15131001)
Supplement: Supplementary file 1 [file biology-15-01001-s001.zip › Figure S1.pdf]

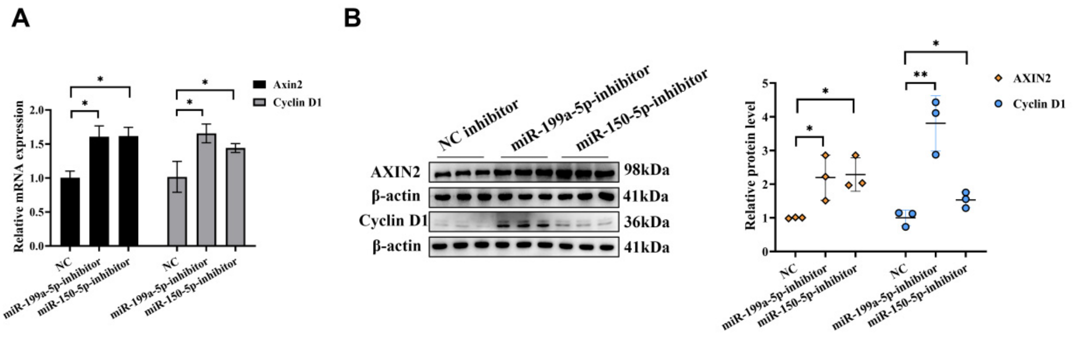

Figure S1. miR-199a-5p and miR-150-5p regulates muscle atrophy through the restoration of key protein in Wnt pathway. (**A**) The relative mRNA expression levels of Axin2 and Cyclin D1 following the transfection of miR-199a-5p or miR-150-5p inhibitors in comparison with NC inhibitors group. (**B**) The relative protein expression levels of Axin2 and Cyclin D1 following the transfection of miR-199a-5p or miR-150-5p inhibitors in comparison with NC inhibitors group. All results are shown as mean±SD. * $p < 0.05$ and ** $p < 0.01$ indicate a statistically significant change relative to the NC inhibitors group.
