# Supplementary material for: Aerobic Exercise Preserves Skeletal Muscle Function in Middle-Aged Mice Through the miR-150-5p/miR-199a-5p–Wnt/FZD4 Signaling Pathway
Source: Biology (Basel). 2026 Jun 25;15(13):1001. doi: 10.3390/biology15131001 (PMC13359755; doi:10.3390/biology15131001)
Supplement: Supplementary file 1 [file biology-15-01001-s001.zip › Supplementary File S1 WB Raw Data/WB Source Data.pptx]

## Slide 1
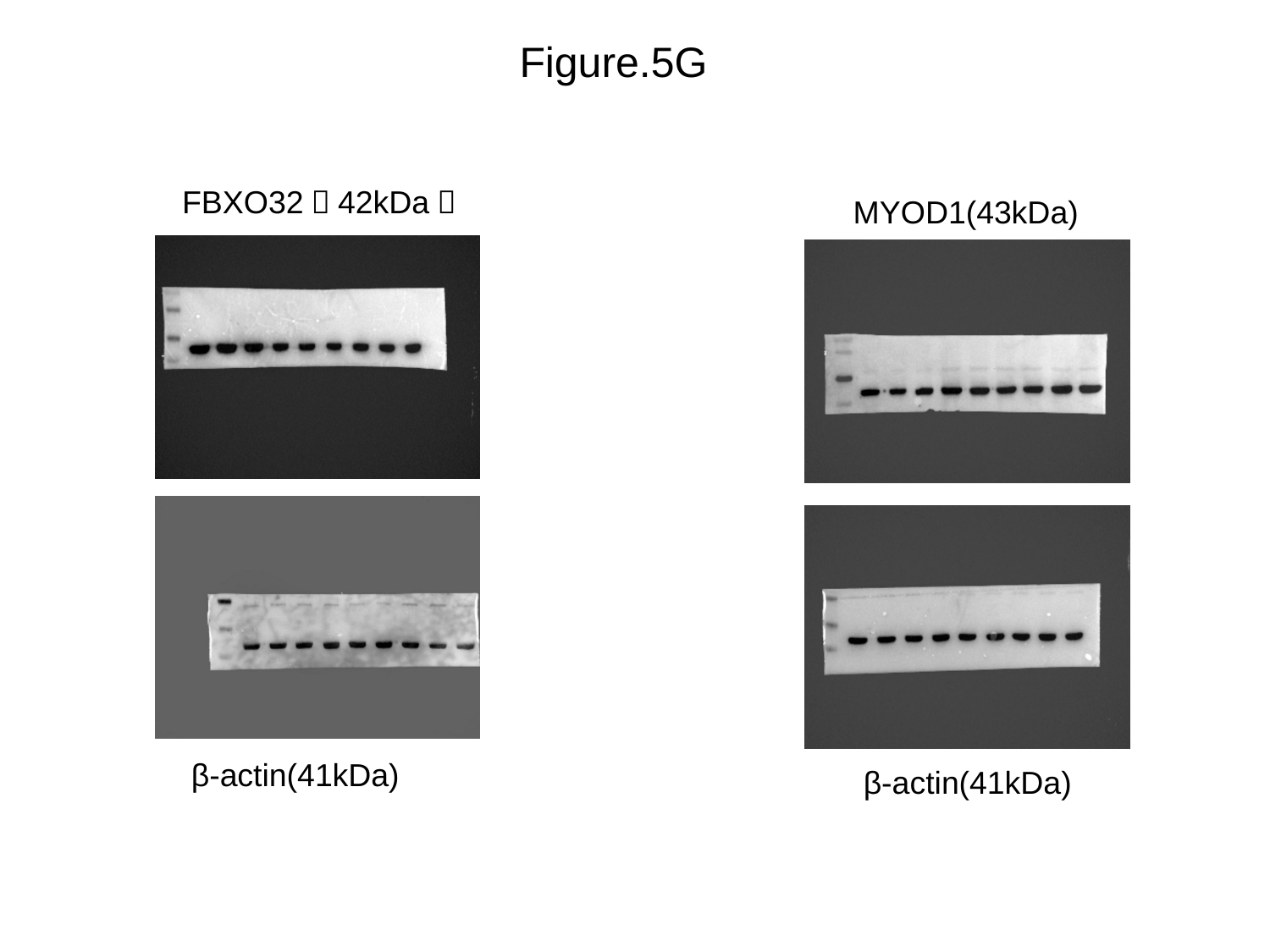

Figure.5G
FBXO32（42kDa）
MYOD1(43kDa)
β-actin(41kDa)
β-actin(41kDa)

## Slide 2
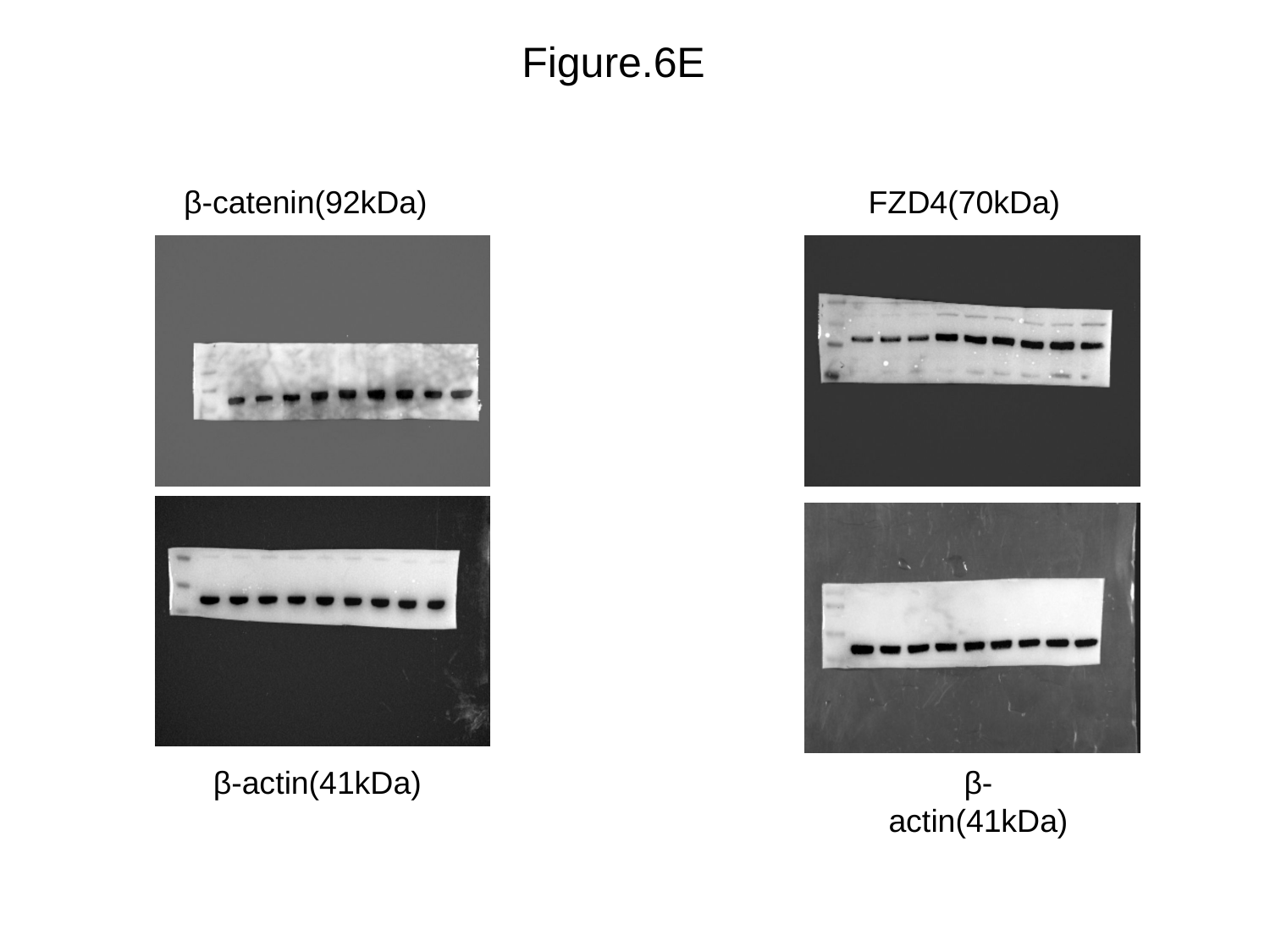

Figure.6E
β-catenin(92kDa)
FZD4(70kDa)
β-actin(41kDa)
β-actin(41kDa)

## Slide 3
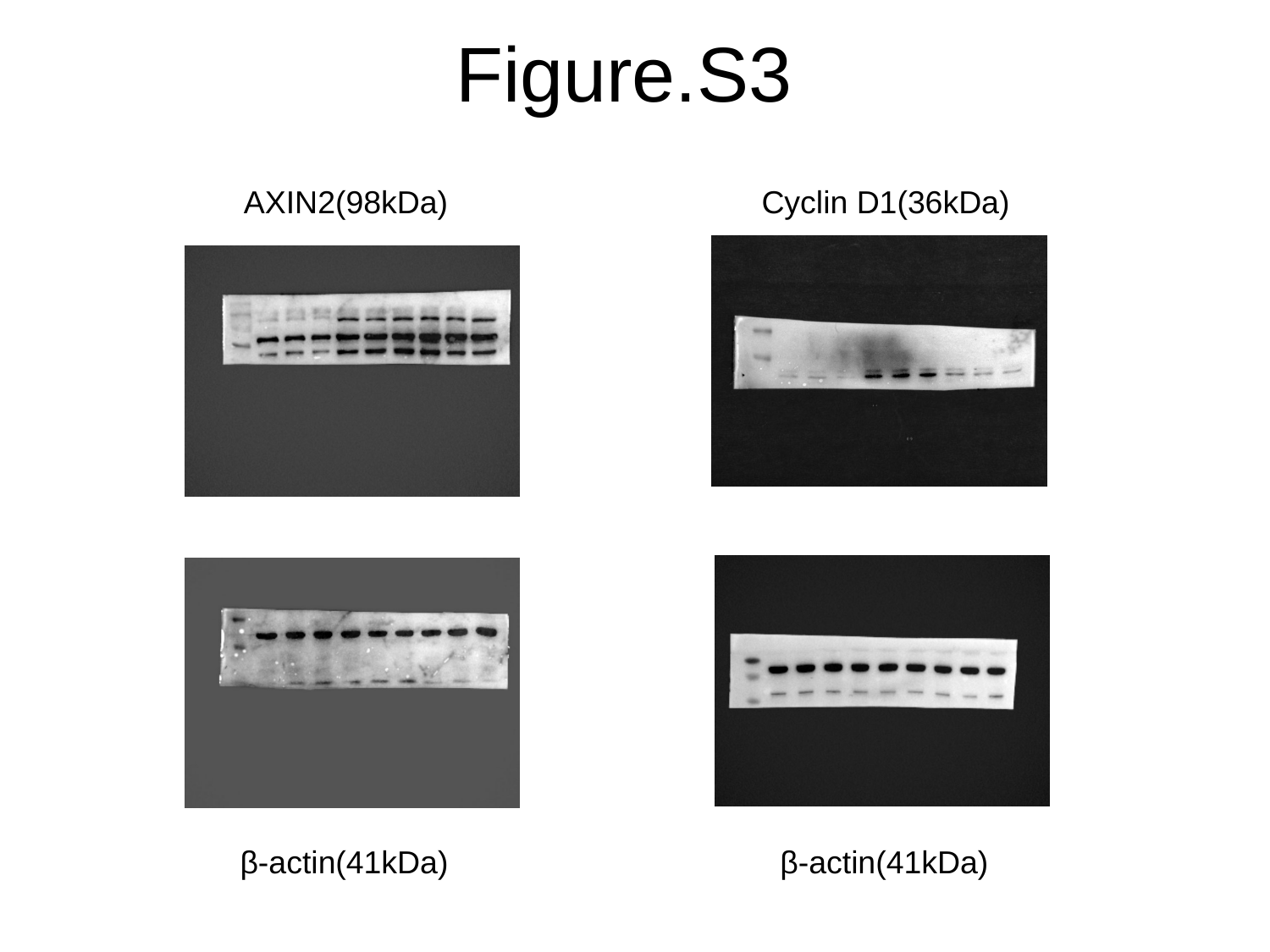

# Figure.S3
AXIN2(98kDa)
Cyclin D1(36kDa)
β-actin(41kDa)
β-actin(41kDa)
