# Supplementary material for: Aerobic Exercise Preserves Skeletal Muscle Function in Middle-Aged Mice Through the miR-150-5p/miR-199a-5p–Wnt/FZD4 Signaling Pathway
Source: Biology (Basel). 2026 Jun 25;15(13):1001. doi: 10.3390/biology15131001 (PMC13359755; doi:10.3390/biology15131001)
Supplement: Supplementary file 1 [file biology-15-01001-s001.zip › Table S1.pdf]

Table S1

Primer sequence for qRT-PCR.

Gene name	Accession numbers	Primer sequence(5'to 3')	Fragments size (bp)
GAPDH-RT-F	NM_001411840	GGCCTCCAAGGAGTAAGAAA	141
GAPDH-RT-R	NM_001411840	GCCCCTCCTGTTATTATGG	
β-actin-RT-F	NM_007393	TCGAGTCGCGTCCACC	157
β-actin-RT-R	NM_007393	GGGAGCATCGTCGCCC	
Atrogin-1-RT-F	NM_026346	GAGCGCCATGGATACTGTACT	113
Atrogin-1-RT-R	NM_026346	GCTATCAGCTCCAACAGCCTTA	
MyoD1-RT-F	NM_010866	CTGCTCTGATGGCATGATGGAT	152
MyoD1-RT-R	NM_010866	CTATGCTGGACAGGCAGTCG	
MyoG-RT-F	NM_031189	GGTCCCAACCCAGGAGATCAT	136
MyoG-RT-R	NM_031189	AGTTGGGCATGGTTTCGTCT	
FZD4-RT-F	NM_008055	TTCCTTTGTTCGGTTTATGTGCC	112
FZD4-RT-R	NM_008055	CTCTCAGGACTGGTTCACAGC	
β-catenin-RT-F	NM_007614	ATGGACTGCCTGTTGTGGTT	115
β-catenin-RT-R	NM_007614	AAAGGCGCATGATTTGCTGG	
AXIN2-RT-F	NM_015732	ACCGCGAGTGTGAGATCC	114
AXIN2-RT-R	NM_015732	GGTGGCTGGTGCAAAGAC	
Cyclin D1-RT-F	NM_007631	CCTGGAGCCCTTGAAGAAGA	67
Cyclin D1-RT-R	NM_007631	CTTAGAGGCCACGAACATGC	
U6-RT-F		CAGCACATATACTAAAATTGGAACG	76
U6-RT-F		ACGAATTTGCGTGTCATCC	
miR-150-5p-RT-F		CAGTATTCTCTCCCAACCCTTGTA	67
miR-150-5p-RT-R		TATGGTTTTGACGACTGTGTGAT	
miR-199a-5p-RT-F		CCGAGACCCAGTGTTCAGACTAC	71
miR-199a-5p-RT-R		CAGTGCGTGTCGTGGAGT	
