# Supplementary figures and images for: Aerobic Exercise Preserves Skeletal Muscle Function in Middle-Aged Mice Through the miR-150-5p/miR-199a-5p–Wnt/FZD4 Signaling Pathway
Source: Biology (Basel). 2026 Jun 25;15(13):1001. doi: 10.3390/biology15131001 (PMC13359755; doi:10.3390/biology15131001)

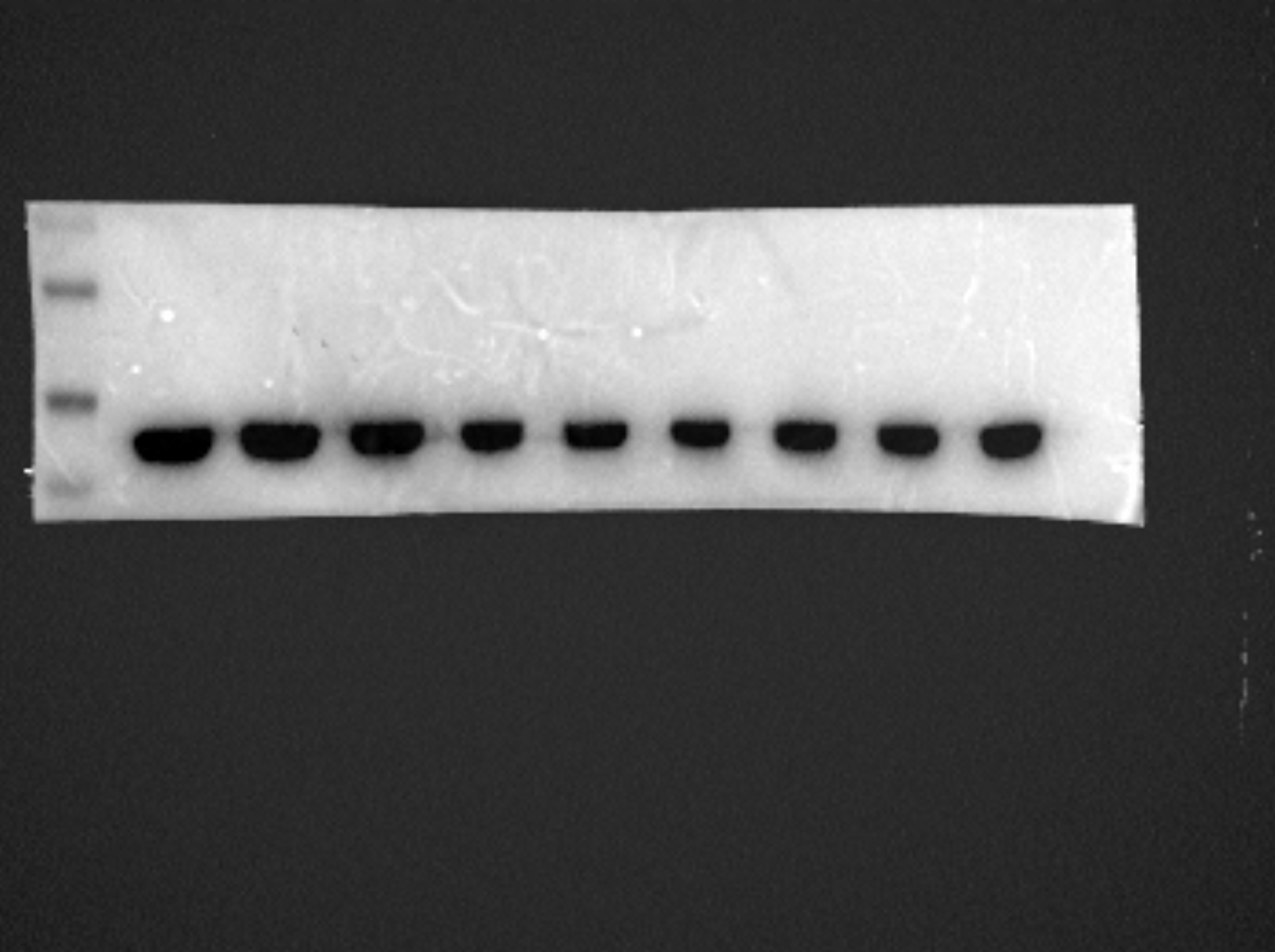

Supplement: Supplementary file 1 [file biology-15-01001-s001.zip › Supplementary File S1 WB Raw Data/5G_FBXO32_full_blot.tif]

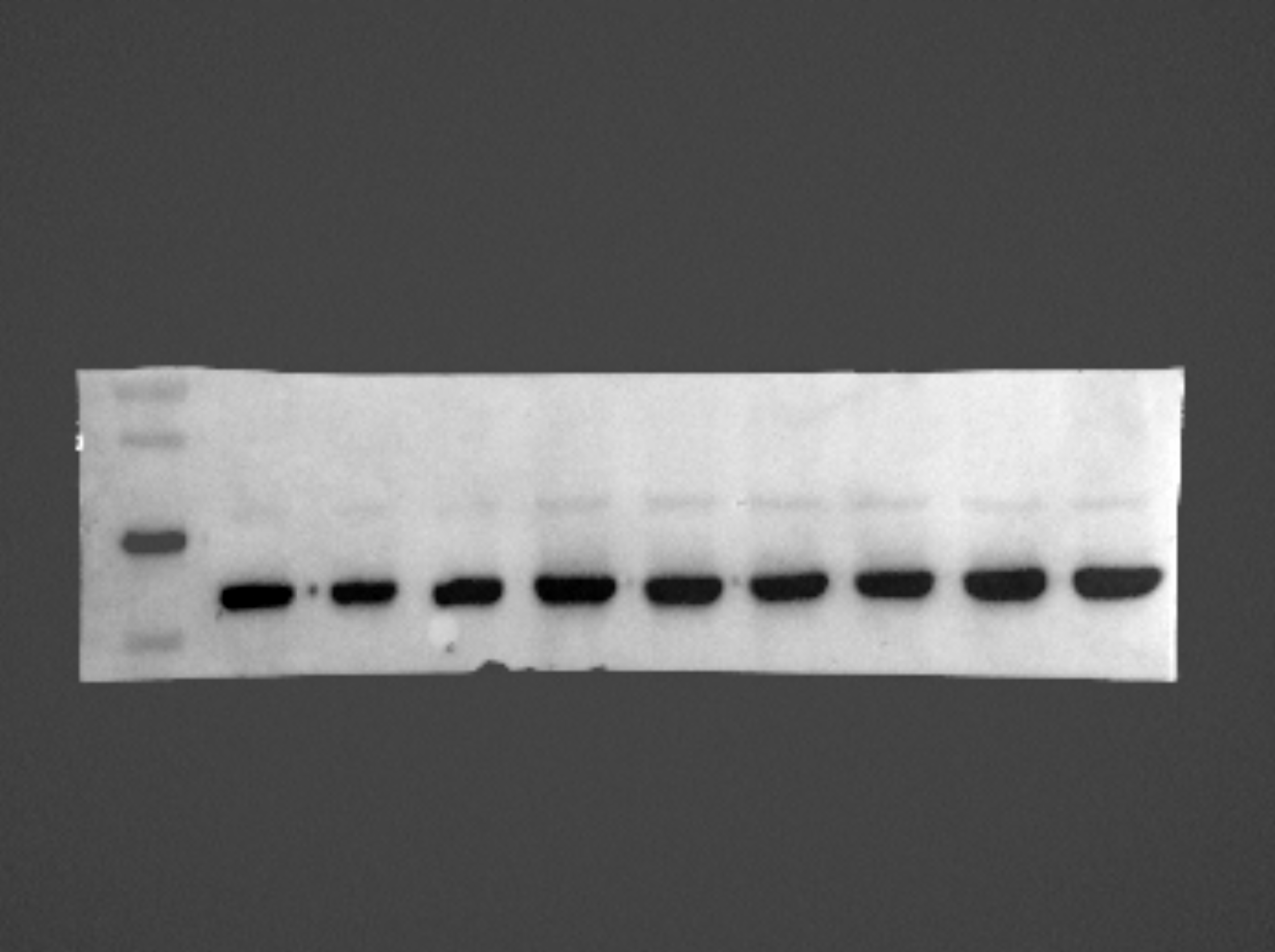

Supplement: Supplementary file 1 [file biology-15-01001-s001.zip › Supplementary File S1 WB Raw Data/5G_MYOD1_full.tif]

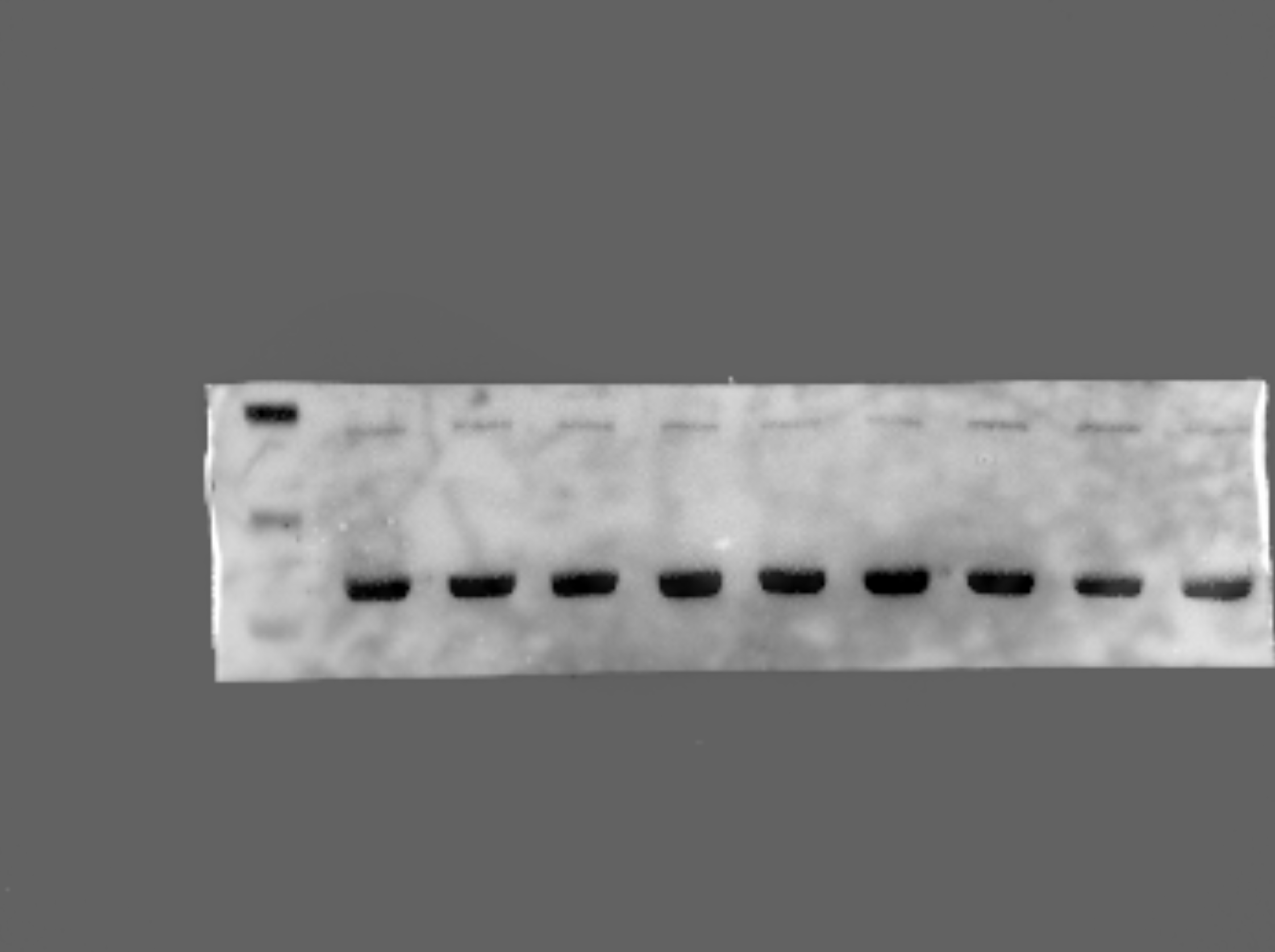

Supplement: Supplementary file 1 [file biology-15-01001-s001.zip › Supplementary File S1 WB Raw Data/5G_a┬-actin-1_full.tif]

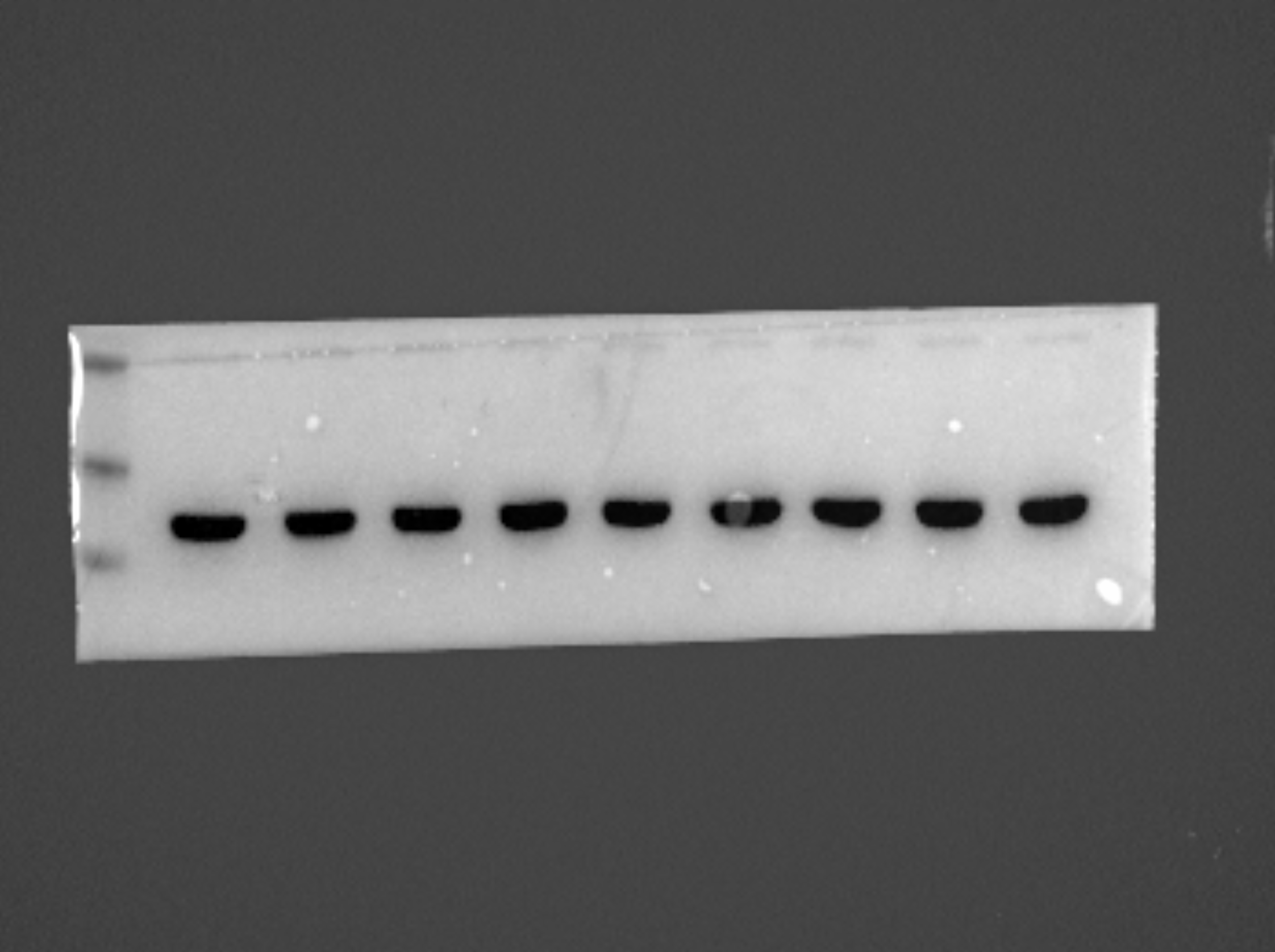

Supplement: Supplementary file 1 [file biology-15-01001-s001.zip › Supplementary File S1 WB Raw Data/5G_a┬-actin-2_full.tif]

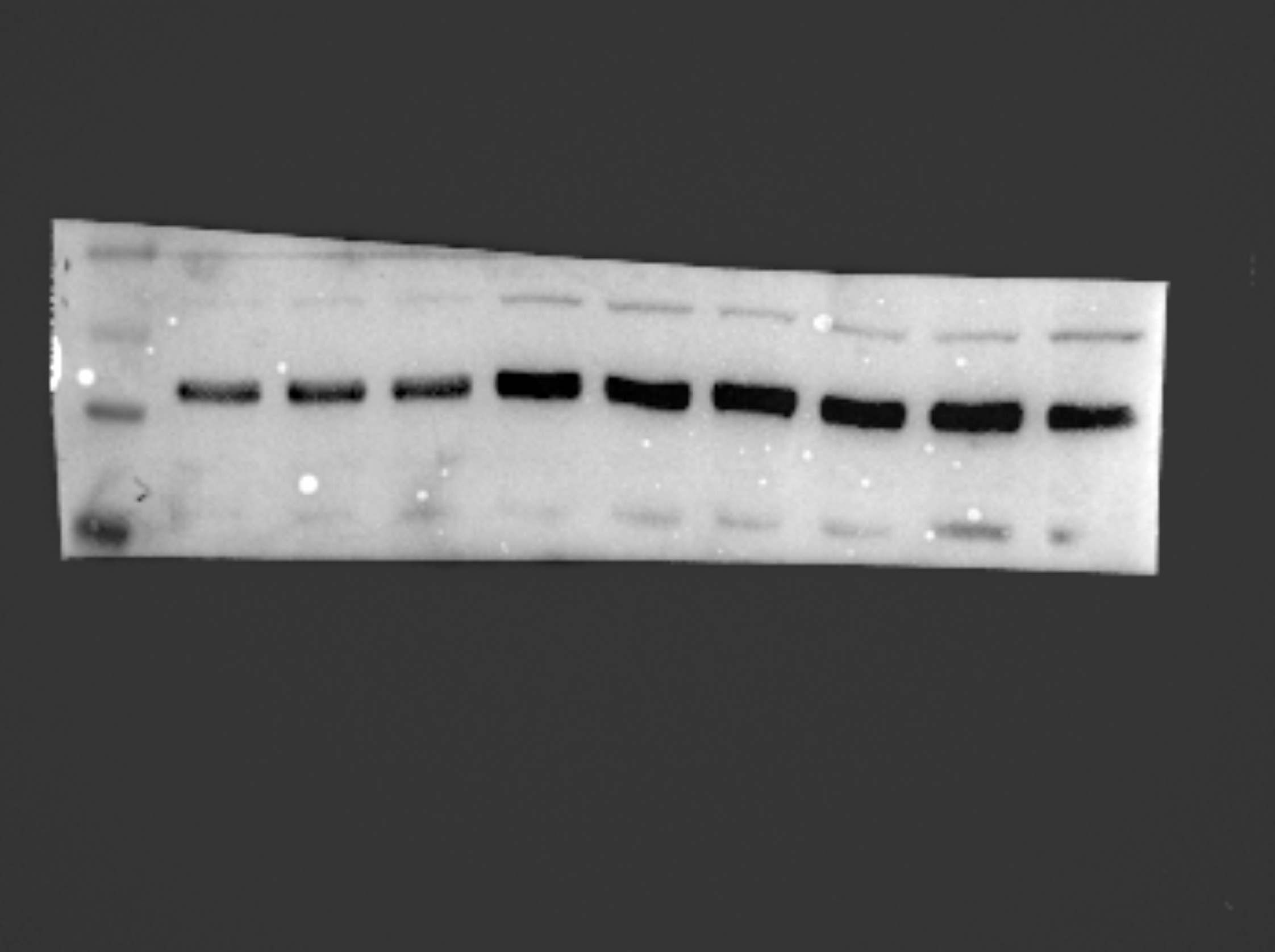

Supplement: Supplementary file 1 [file biology-15-01001-s001.zip › Supplementary File S1 WB Raw Data/6E_FZD4-_full.tif]

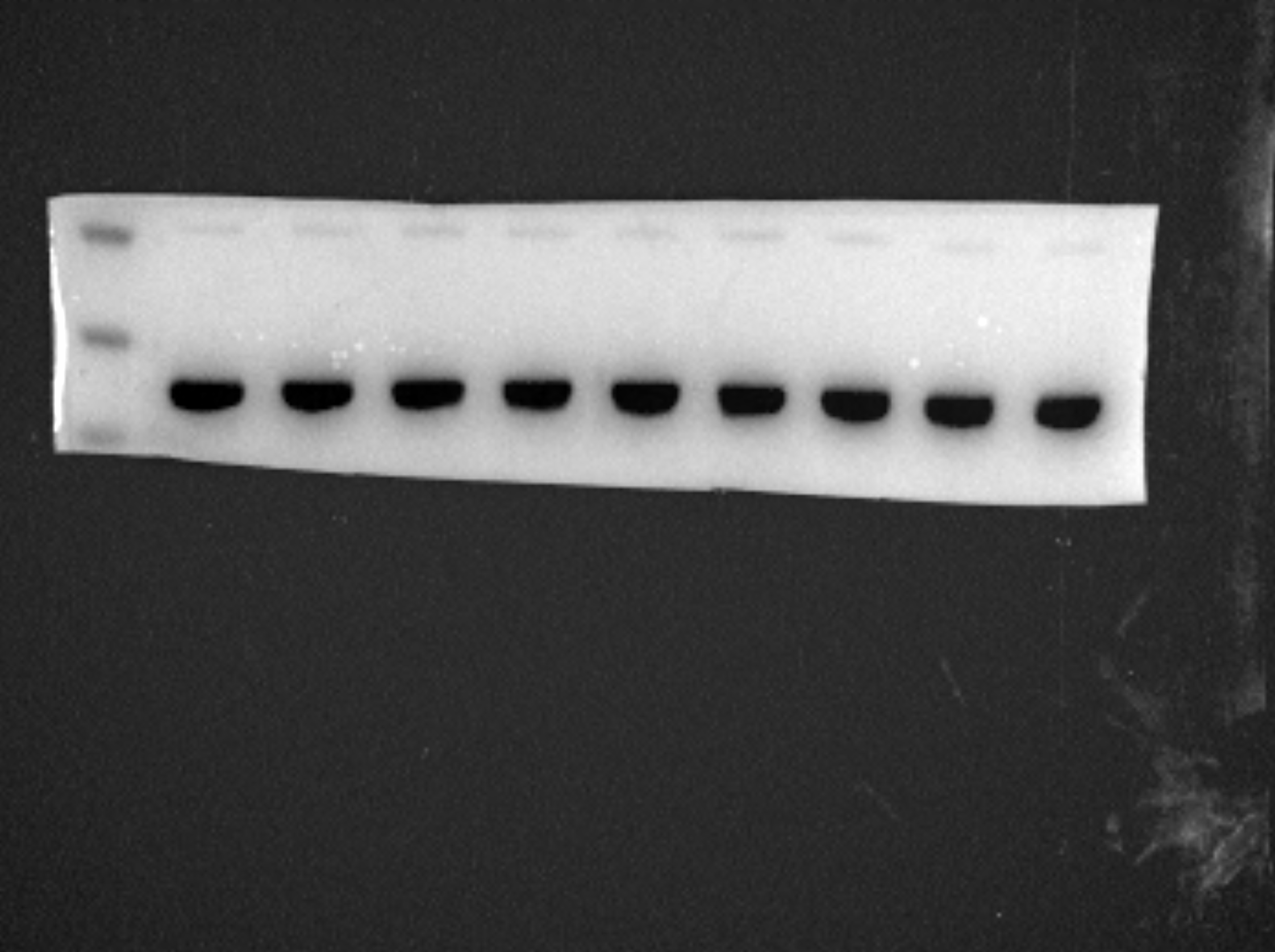

Supplement: Supplementary file 1 [file biology-15-01001-s001.zip › Supplementary File S1 WB Raw Data/6E_a┬-actin-1_full.tif]

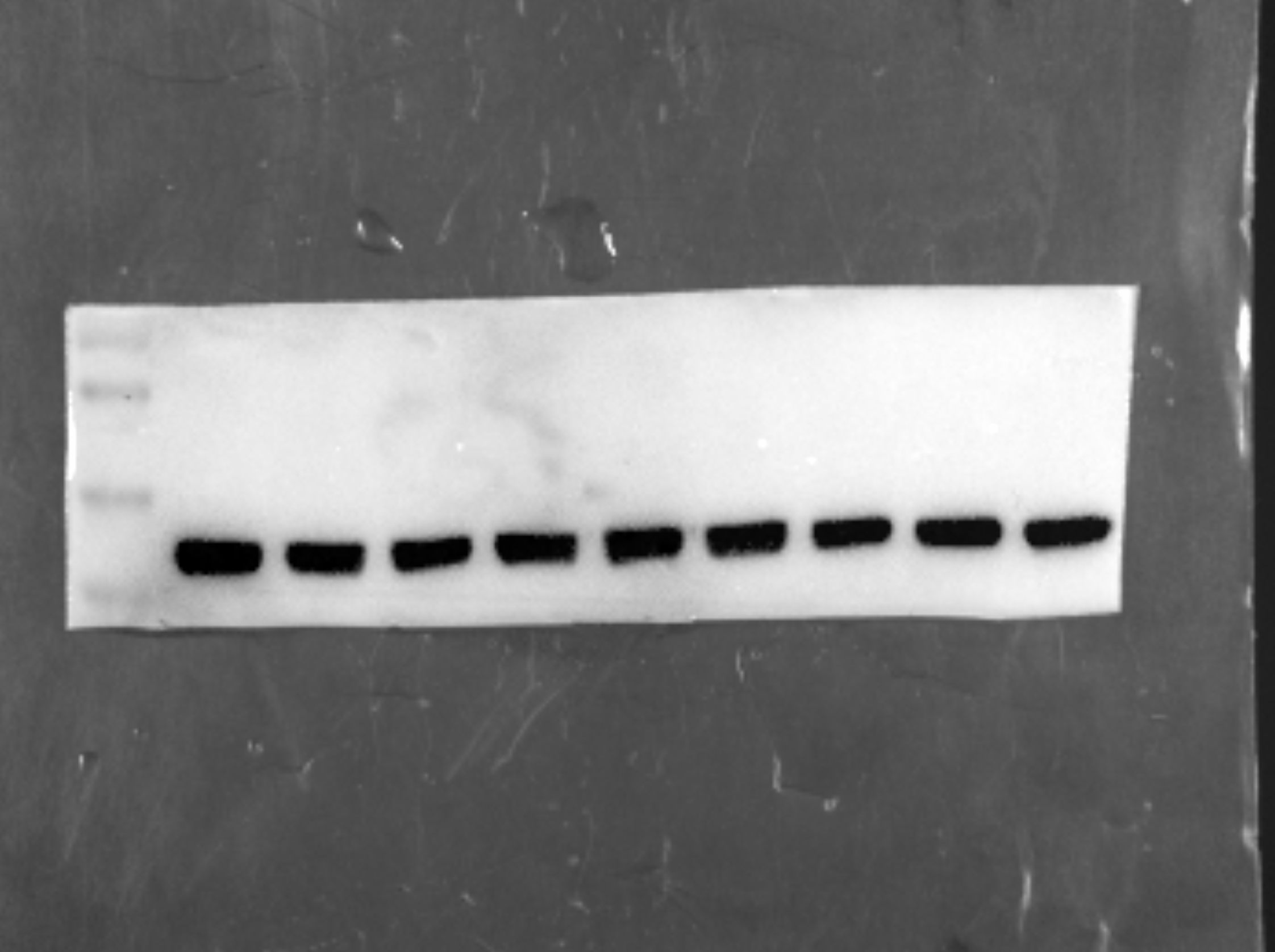

Supplement: Supplementary file 1 [file biology-15-01001-s001.zip › Supplementary File S1 WB Raw Data/6E_a┬-actin-2_full.tif]

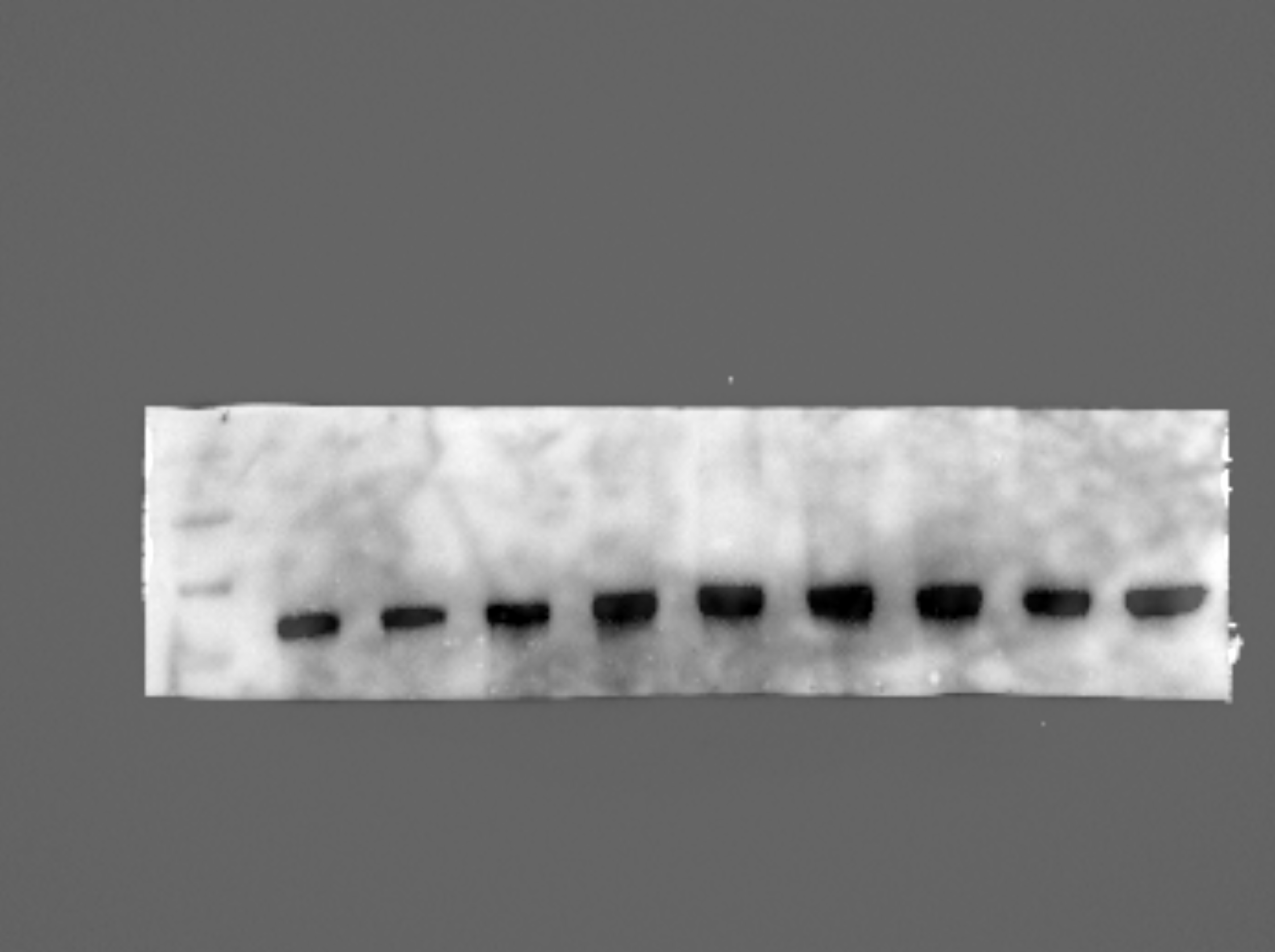

Supplement: Supplementary file 1 [file biology-15-01001-s001.zip › Supplementary File S1 WB Raw Data/6E_a┬-catenin-_full.tif]

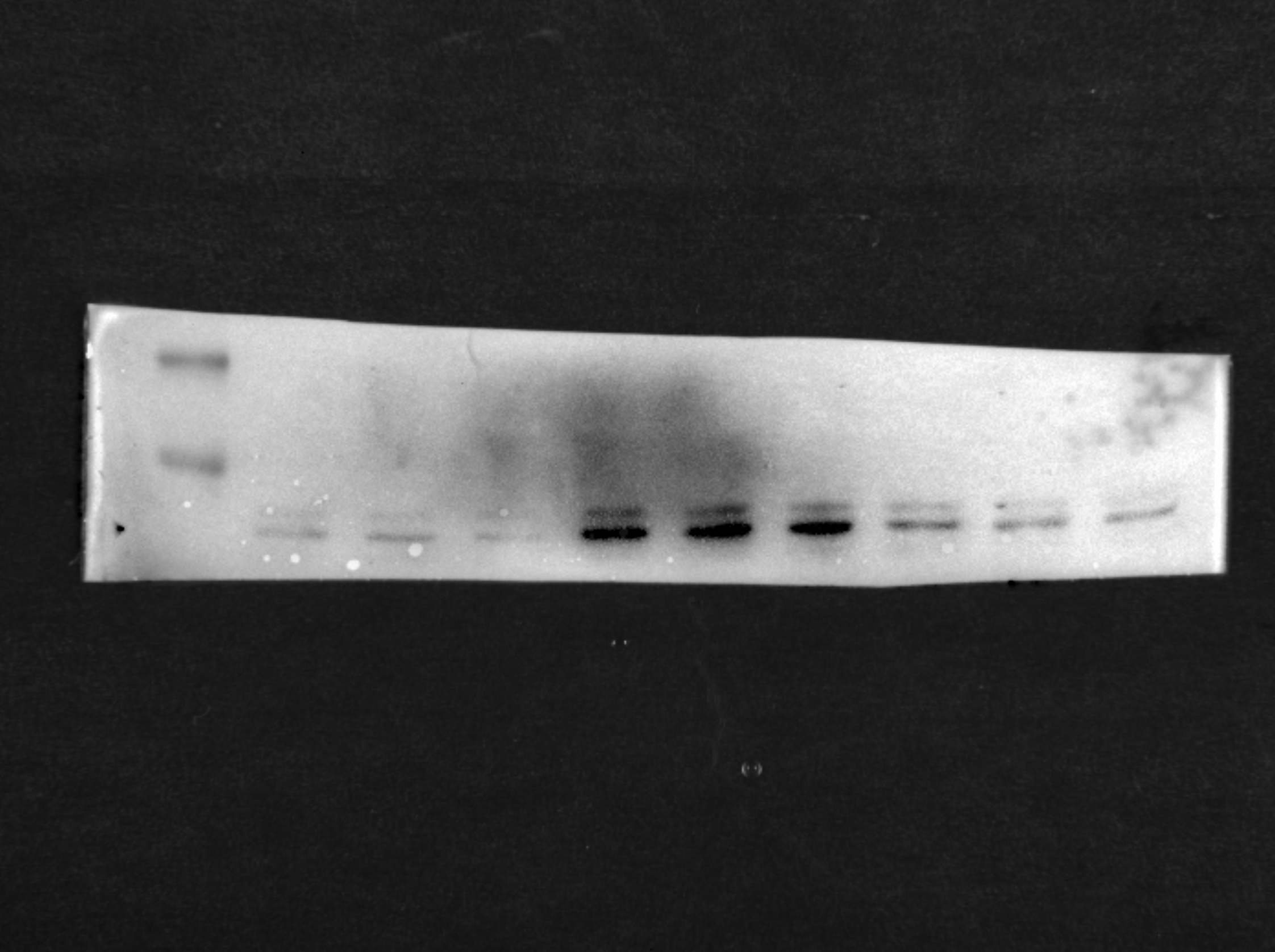

Supplement: Supplementary file 1 [file biology-15-01001-s001.zip › Supplementary File S1 WB Raw Data/M-CYDIND1+CYDIND1.tif]

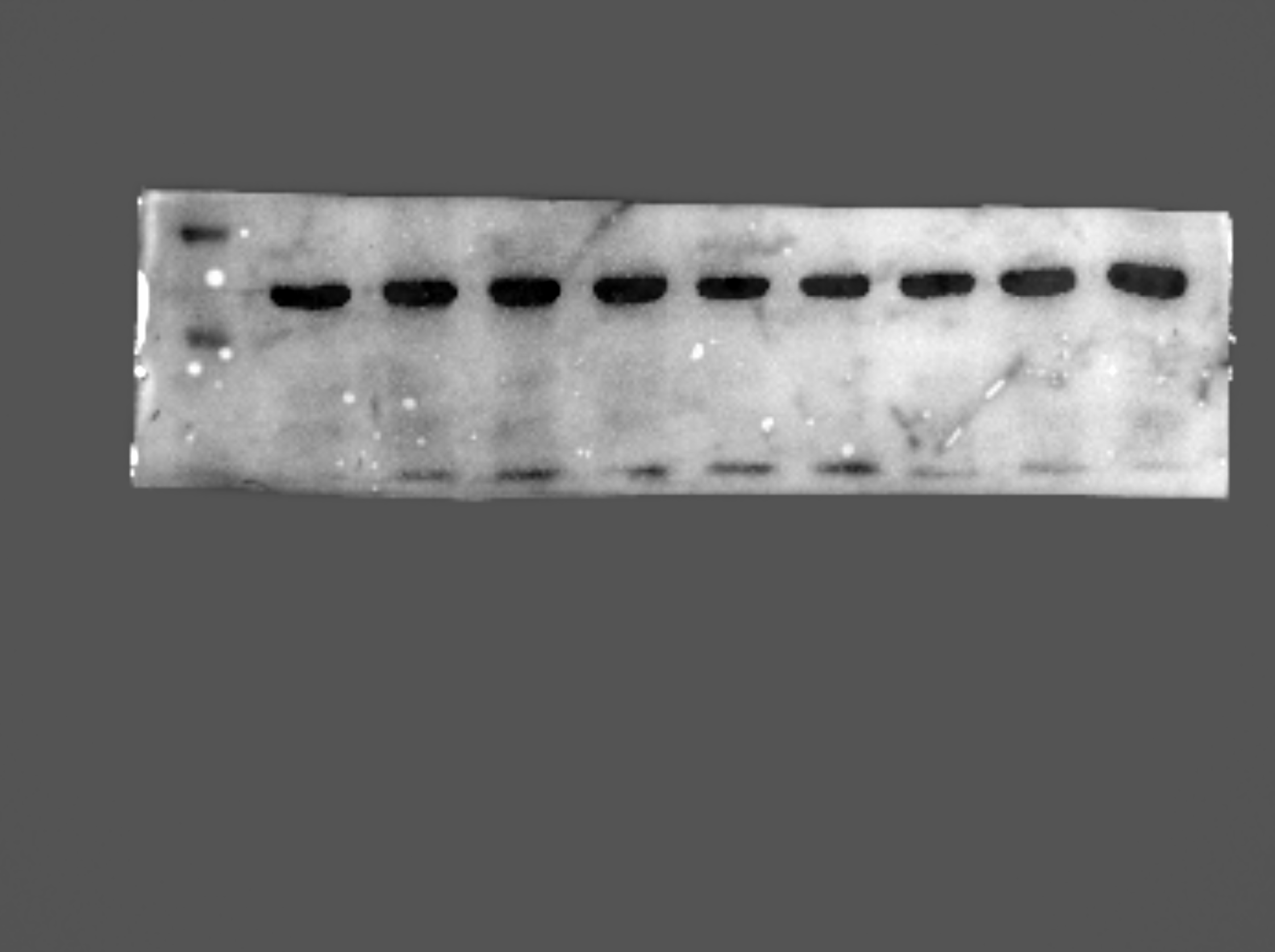

Supplement: Supplementary file 1 [file biology-15-01001-s001.zip › Supplementary File S1 WB Raw Data/S3_actin_1 (2).tif]

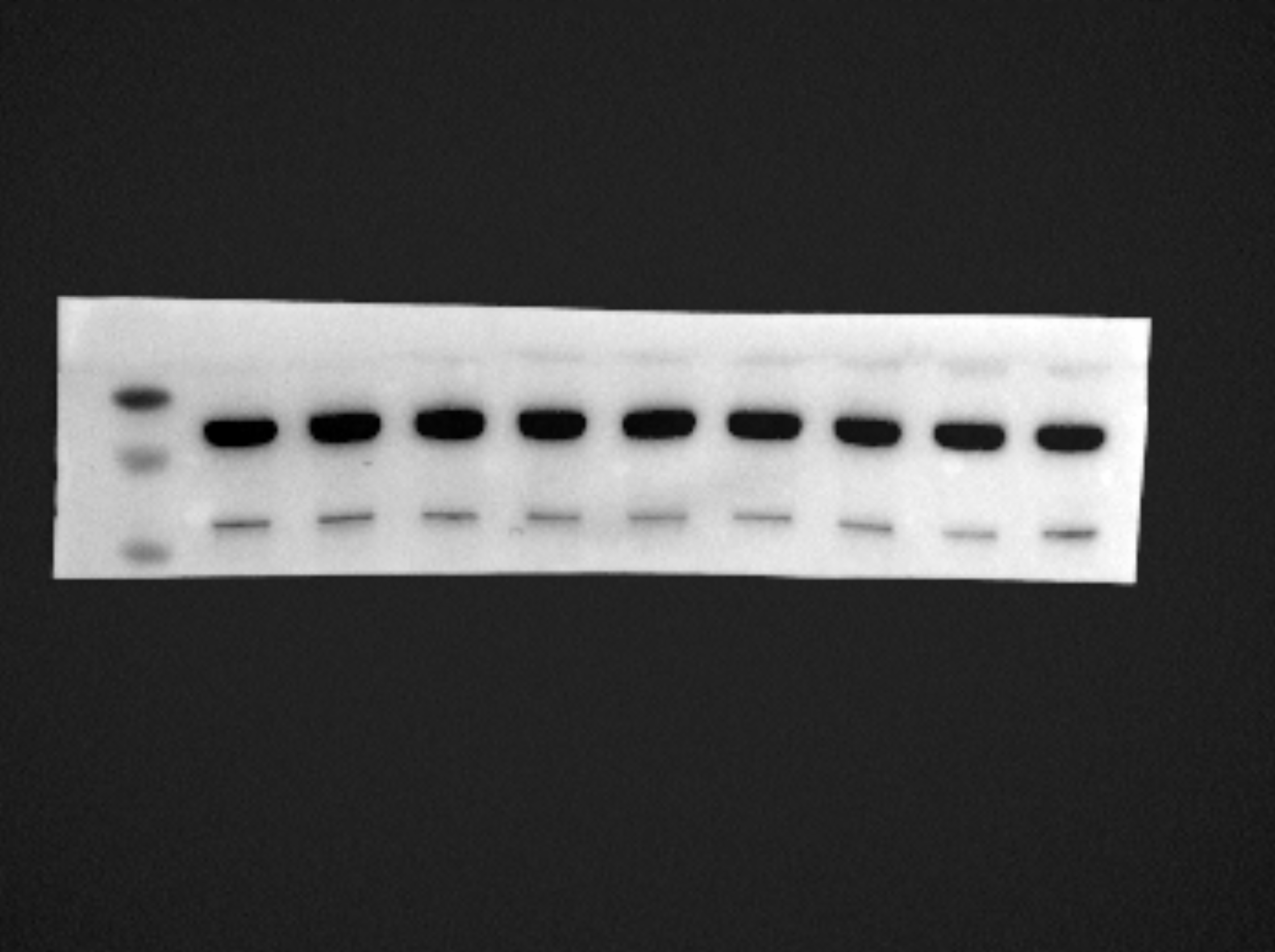

Supplement: Supplementary file 1 [file biology-15-01001-s001.zip › Supplementary File S1 WB Raw Data/S3_actin_2.tif]

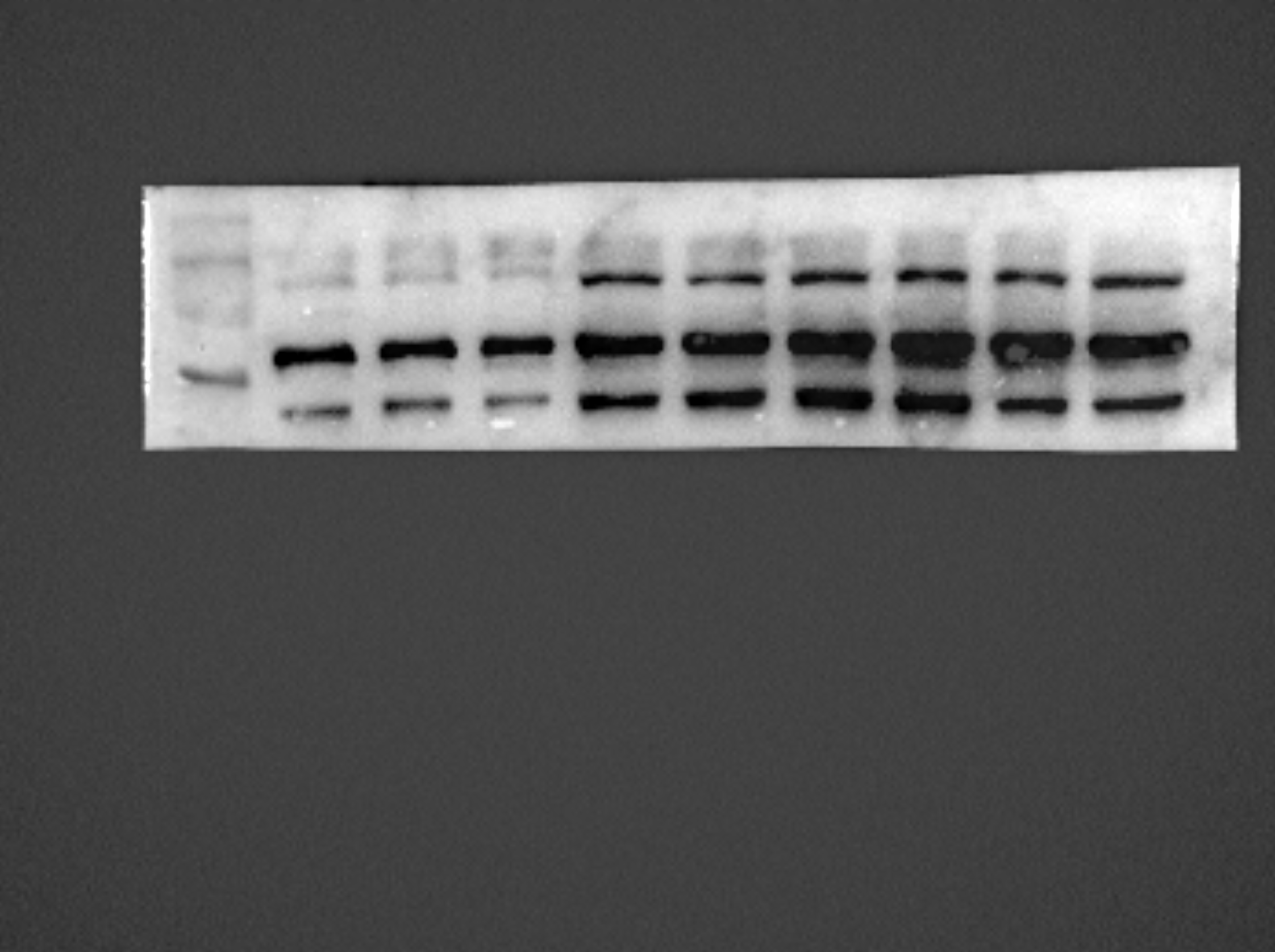

Supplement: Supplementary file 1 [file biology-15-01001-s001.zip › Supplementary File S1 WB Raw Data/S3_AXIN2_full_blot.tif]
